# Identification and validation of SUMOylation-related key genes for osteoarthritis through integration of single-cell, bulk RNA sequencing and animal model experiments

**DOI:** 10.3389/fmed.2026.1779874

**Published:** 2026-03-31

**Authors:** Ansong Liu, Yanlin Yang, Jie Xiang, Yiheng Hu, Jifa Yang, Yu Zhang, Yue Deng, Yong Chen

**Affiliations:** 1 Department of Orthopedics, The First Affiliated Hospital, University of South China, Hengyang, Hunan, China; 2 Department of Spinal Surgery, The First Affiliated Hospital, University of South China, Hengyang, Hunan, China

**Keywords:** key cells, key genes, osteoarthritis, single-cell RNA sequencing, SUMOylation

## Abstract

**Background:**

Osteoarthritis (OA) existed as a degenerative arthropathy that involved the cartilage and many of its surrounding tissues. SUMOylation was an important post-translational modification. Some studies had confirmed that SUMOylation was associated with the occurrence of OA. However, there were relatively few related studies, and the explicit action mechanism of SUMOylation-related genes (SRGs) in OA remained unclear. The objective of this research was to detect and verify key genes related to SRGs in OA.

**Methods:**

Firstly, GSE51588 (GPL13497), GSE55235 (GPL96) and GSE152805 and SRGs were from public databases. Subsequently, key genes were determined by differential expression analysis, PPI network, machine learning, expression analysis and ROC curves. Following this, using key genes, functional enrichment, immune infiltration, and drug prediction were implemented. Meanwhile, the identification of key cells was achieved by conducting single-cell RNA sequencing (scRNA-seq) analysis. Finally, a rat model (three OA and three sham samples) was established, and the expressions of key genes were verified.

**Results:**

NUP98 and TOP1 were identified as key genes. Later, the two key genes were co-enriched in multiple pathways, like “ribosome.” Significant differences in 14 key immune cells (like monocytes) were observed between the OA and control groups. Furthermore, the drug-key gene network indicated that eight drugs (like luteolin) were associated with two key genes. Moreover, by scRNA-seq analysis, homeostatic chondrocytes (HomCs) were recognized as key cells. During the differentiation of HomCs, the expression level of both NUP98 and TOP1 first increased and then remained stable. Finally, the expression of two key genes at both gene and protein levels were notably elevated in the sham group.

**Conclusion:**

In this study, two key genes and one key cell type were identified, which provided new directions for progress in OA research.

## 1 Introduction

Osteoarthritis (OA), the most common degenerative arthropathy globally, is marked by gradual cartilage breakdown, subchondral bone restructuring, and bone spur development (1). Epidemiological studies estimate that approximately 240 million individuals globally suffer from symptomatic OA (2). Driven by population aging, obesity epidemics, and increasing joint trauma, OA prevalence is projected to rise substantially over the next three decades (3). Current clinical management faces significant limitations: early-stage interventions relying primarily on non-steroidal anti-inflammatory drugs (NSAIDs) merely afford symptomatic alleviation without halting disease progression, while end-stage disease necessitates total joint arthroplasty—a procedure burdened by substantial costs (>USD 20,000 per case) and risks of surgical complications (e.g., 3–5% infection rate) (4). Thus, the development of reliable diagnostic biomarkers for early intervention, alongside novel therapeutic strategies with disease-modifying effects, favorable safety profiles, and cost-effectiveness, constitutes an urgent unmet clinical imperative.

The present investigation posits that osteoarthritis (OA) arises from dysregulation of the “cartilage-subchondral bone-synovium” axis, which operates as a functionally integrated triad rather than discrete anatomical entities. Mechanistically: (i) progressive degeneration of articular cartilage constitutes the principal pathological terminus (5); (ii) maladaptive remodeling of subchondral bone provides both biomechanical and biochemical impetus for disease advancement (6); and (iii) synovial inflammation sustains a catabolic milieu via persistent cytokine elaboration (7). Intertissue crosstalk-mediated by intercellular communication, paracrine signaling cascades, and metabolic feedback circuits-converges to amplify degenerative processes within the joint. Moreover, senescent mesenchymal stem cells (MSCs) accumulate in cartilage, synovium and subchondral bone of OA joints, and their secreted senescence-associated secretory phenotype (SASP) exerts synergistic pathological effects on different joint tissues, including promoting cartilage fibrosis, impairing cartilage regeneration and inducing subchondral bone sclerosis (8–10); multinucleated giant cells in synovium are also associated with subchondral osteoclast activation and cartilage damage, further verifying the non-independent pathological changes among distinct joint compartments (11).

Post-translational modifications (PTMs) refer to covalent modifications to proteins post-translationally, dynamically regulate protein stability, localization, and interactions, thereby governing cellular homeostasis (12). Among diverse PTMs—including phosphorylation, acetylation, and ubiquitination—SUMOylation, a ubiquitin-like modification, uniquely orchestrates nuclear processes like transcriptional regulation and DNA repair. Dysregulated PTMs, particularly SUMOylation, are implicated in cancer, neurodegeneration, and metabolic disorders through aberrant signaling cascades (13, 14). SUMOylation, a ubiquitin-like PTM, regulates fundamental nuclear processes—containing gene expression, cell cycle progression, DNA damage response, and proteostasis—through substrate-specific modification of functional proteomes. This modification stabilizes target proteins and promotes oncogenic signaling, exemplified by its activation of AKT to accelerate cell proliferation and carcinogenesis (13). Concurrently, SUMOylation governs neural development and modulates the progression of neurodegenerative conditions such as Parkinson’s disease (14). Emerging evidence suggests SUMOylation regulates chondrocyte metabolism via nucleocytoplasmic transport and protein stabilization, implicating its role in OA progression (15, 16). SUMOylation, a phylogenetically conserved nuclear post-translational modification, underpins inter-organ regulatory networks at the molecular level. Systematic interrogation of SUMOylation-related genes (SRGs) across multiple joint tissues enables elucidation of their pleiotropic functions in osteoarthritis (OA), supporting our trans-tissue investigative hypothesis: pivotal SRGs may orchestrate the above-described conserved signaling cascades and act in concert within the “cartilage-subchondral bone-synovium” axis to propel the holistic joint pathology characteristic of OA.

Single-cell RNA sequencing (scRNA-seq) revolutionizes transcriptomic analysis by resolving gene expression heterogeneity across individual cells, enabling the identification of rare cell states and dynamic transitions undetectable in bulk sequencing (2). By overcoming the limitations of cellular heterogeneity, scRNA-seq precisely identifies rare cell subpopulations, maps cellular developmental trajectories, and reconstructs interaction networks, providing a “cellular microscope” for mechanistic insights into complex diseases (17). Recent studies have demonstrated the application of scRNA-seq in analyzing knee joint cartilage and meniscal tissues from OA patients, revealing pathogenic cell subpopulations (5, 18). scRNA-seq has reshaped the pathological understanding of OA from a cellular ecological perspective, offering unique advantages such as single-cell resolution, dynamic tracking capabilities, and the integration of multi-omics data. These features make scRNA-seq an indispensable tool for deciphering the network-level regulatory mechanisms of SUMOylation.

In this study, we identified key genes associated with OA by leveraging transcriptomic datasets and synovial receptor genes (SRGs) through a series of bioinformatics approaches. Functional enrichment analysis, immune infiltration analysis, molecular regulatory network analysis, and drug prediction were conducted to analyze the molecular regulatory mechanisms engaged in these key genes. To further explore OA at the cellular level, we incorporated single-cell datasets. Based on these datasets, we identified key cells associated with the key genes. Through cell communication analysis, we explored interactions between cells and utilized pseudotime analysis to clarify the differentiation status of key cells and the dynamic changes in key gene expression during differentiation. Finally, we validated the expression and distribution of key genes using OA animal models. Overall, these findings afford a strong theoretical groundwork for effective diagnostic and therapeutic approaches in OA.

## 2 Materials and methods

### 2.1 Data collection

GSE51588 (GPL13497) (19), GSE55235 (GPL96) (20), GSE117999 (GPL20844) (21), GSE82107 (GPL570) (22) and GSE152805 (GPL24676) (23) were obtained from the GEO database.^1^ Specifically, GSE51588 (training set) consisted of subchondral bone tissue samples from 40 osteoarthritis (OA) patients (as the OA group) and 10 non-OA (as the control group), while GSE55235 (validation set) contained synovial tissue samples from 10 OA patients and 10 healthy controls, respectively. Meanwhile, single-cell dataset GSE152805 consisted of six OA patients’ cartilage samples. GSE 117999 identified gene transcripts differentially expressed in cartilage tissues obtained from 12 patients with osteoarthritis and from 12 patients without osteoarthritis (arthroscopic partial meniscectomy). GSE82107 included 10 microarrays of end-stage osteoarthritis (OA) synovial biopsies and seven microarrays of synovial biopsies from individuals without a joint disease. Furthermore, 194 SUMOylation-related genes (SRGs) were acquired via literature review (24) (Supplementary Table S1).

### 2.2 Differential expression analysis and function analysis

In GSE51588, the genes showing differential expression (DEGs) between OA samples and control samples (OA vs. control) were determined via the “limma” package (v 3.54.0) (25), the original *p*-value was calculated using the moderated t-statistic, and multiple testing was corrected by the Benjamini–Hochberg method (FDR <0.05) (|log_2_FoldChange(FC)| >0.5, adj. *p* < 0.05). Meanwhile, to have a full grasp of the distribution state of DEGs, the volcano map was plotted via the “ggplot2” package (v 3.4.1) (26), and the DEGs were sorted according to log_2_FC in descending order, with top 10 regulated genes in the volcano plot were labeled. The heatmap was produced via the “pheatmap” package (v 1.0.12) (27) to display all DEGs.

Then, the “VennDiagram” package (v 1.7.3) (28) was utilized to determine candidate genes by intersecting DEGs and SRGs. Ultimately, GO and KEGG analyses were applied to reveal pathways (adj. *p* < 0.05) of candidate genes via “clusterProfiler” (v 4.2.2) (29). All analyses were corrected for *p*-values using the Benjamini–Hochberg (BH) method.

### 2.3 Identification of feature genes

Following this, based on candidate genes, a PPI network (interaction scores ≥0.4) was created by STRING site,^2^ drawn via Cytoscape software (v 3.5.2) (30). Meanwhile, the CytoHubba plugin in Cytoscape software (v 3.5.2) was utilized to conduct four algorithms, including Density of Maximum Neighborhood Component (DMNC), EPC (Edge-Point Classification), Betweenness, and Stress. Finally, the core genes were ascertained through intersecting the top 20 genes from each of the four algorithms.

Subsequently, to identify the most important genes among the core genes, machine learning was performed on all samples in GSE51588. Based on the core genes, the “glmnet” package (v 4.1.2) (31) was employed for LASSO regression analysis. 10-fold cross-validation was employed to precisely identify the feature genes (optimal lambda and coefficients ≠ 0).

### 2.4 Identification of key genes

Based on feature genes, expression analysis in GSE51588 and GSE55235 was independently conducted, and the genes demonstrating a marked distinction between OA and control groups (*p* < 0.05) and consistent expression trend in two datasets, were chosen as candidate key genes.

Then, based on these candidate key genes, the ROC curves were mapped on all samples in GSE51588 and GSE55235 via the “pROC” package (v 1.18.0) (32), respectively. Genes with area under the curve (AUC) values >0.7 in two datasets were selected as key genes. To check if key genes’ consistent expression across tissues happened by chance, we used random gene sets. For each set, we calculated how many genes were strongly linked to the key genes (|*r*| > 0.3, *p* < 0.05). The empirical *p*-value is the fraction of random sets showing stronger consistency than the key genes. Cross-tissue expression consistency analysis: Public OA cartilage transcriptomic datasets (GSE117999, GSE82107; paired OA vs. normal cartilage) were integrated with existing subchondral bone (GSE51588) and synovium (GSE55235) datasets (Control:OA = 54:53). Expression differences of key genes between OA and control groups across three tissues were analyzed using box plots, with Wilcoxon test for statistical validation.

### 2.5 Building of nomogram

To further evaluate the overall predictive capacity of key genes, within the GSE51588 dataset, a predictive nomogram was built for key genes via the “rms” package (v 6.5.0) (33). The OA incidence risk was then predicted based on the total score; a higher score signified a greater risk of OA. To measure the nomogram’s prediction precision, calibration curves were created, the ROC curve was plotted by the “pROC” package (v 1.18.0) (AUC >0.7), and DCA was conducted by the “ggDCA” package (v 1.2).^3^

### 2.6 Gene set enrichment analysis and GeneMANIA

To understand the pathways involved in OA progression of key genes, GSEA was carried out on all samples in GSE51588 (adj. *p* < 0.05). Firstly, gene sets of the human KEGG pathway were retrieved from MSigDB^4^ to serve as a background gene set. Subsequently, Spearman correlation coefficients among key genes and genes in GSE51588 were calculated by the “psych” package (v 2.2.9) (34). Subsequently, all genes were arranged in a descending sequence according to the correlation coefficient, and for each key gene, a list of corresponding related genes was acquired. The “clusterProfiler” package (v 4.2.2) was used for GSEA (*p* < 0.05).

Moreover, the GeneMANIA database^5^ was applied to forecast the functions of key genes and delve into their closely associated functions. To investigate whether key genes are involved in a unified biological mechanism across different joint tissues, we performed Gene Set Enrichment Analysis (GSEA) using the fgsea package (v1.24.0) on their expression profiles from subchondral bone, synovium, and cartilage.

### 2.7 Immune infiltration

To further appraise the variations in immune status in the process of OA progression, all OA and control samples in GSE51588 underwent immune infiltration analysis. The CIBERSORT algorithm was applied to calculate infiltration scores for 22 cell types (35). Meanwhile, the cor function was utilized to conduct Spearman correlation analysis to explore relationships between 22 immune cells, with correlations considered significant at |cor| > 0.3 and *p* < 0.05. Subsequently, the differences in immune cell infiltration between the OA and control samples were analyzed by the Wilcoxon test (*p* < 0.05). Spearman correlation analysis was additionally employed to probe into the relationships among differential immune cells and key genes, where the absolute value of |cor| >0.3 and *p* < 0.05.

### 2.8 Establishment of molecular regulatory networks and drug prediction analysis

The miRTarBase^6^ and miRDB database^7^ were utilized to predict the upstream microRNAs (miRNAs) that interact with key genes. The shared miRNAs among the two databases were obtained through intersection. Moreover, the miRNet database^8^ was used to forecast the long non-coding RNAs (lncRNAs) targeting these shared miRNAs. Finally, on the basis of the aforementioned shared miRNAs and lncRNAs, the lncRNA-miRNA-key gene regulatory network was drawn using Cytoscape software (v 3.5.2).

Meanwhile, in an attempt to identify potential drugs that could regulate key genes, the drugs associated with key genes were searched using the Enrichr database.^9^ And a drug-key gene interaction network was developed and visualized via Cytoscape software (v 3.5.2).

### 2.9 scRNA-seq analysis

The data regarding OA samples sourced from GSE152805 was filtered for scRNA-seq analysis by the “Seurat” package (v 5.0.1) (36). Specifically, the criteria for filtered were that (1) gene expression was observed in fewer than three cells; (2) low-fidelity cells with <200 genes and cells with aberrant high expression containing > 6,000 genes; (3) the total expression measured per cell was <100 and >40,000; (4) cells with mitochondrial percentage >10% (37). The above filtered data were normalized by the NormalizeData function. Then, the FindVariableFeatures function was employed to select the top 2,000 highly variable genes (HVGs). After that, the ScaleData function was applied to scale the data. Then, the JackStrawPlot function was served to contrast the *p*-values distribution of each principal component (PC) (*p* < 0.05), and the principal component analysis (PCA) on the HVGs and PCs was applied by the RunPCA function. The PCA scree plot was visualized by applying the ElbowPlot function, and the number of PCs was picked. Unsupervised clustering analysis of cells was applied by the FindClusters functions (resolution = 0.4) (38–40), and UMAP was applied for dimensionality reduction and to display the clustering results. Furthermore, we annotated the cell clusters via marker genes from the literature (5, 23, 41). The bubble plot depicting different marker gene expression in different cell types was drawn.

### 2.10 Identification of the key cells

In GSE152805, for the purpose of showing how key genes were expressed among all the annotated cell types, UMAP plots were created. Subsequently, a histogram was employed to show the expression of key genes in each cell type, and the cells with the highest expression of all key genes among different cell types were selected as the key cells for subsequent analysis.

### 2.11 Cell communication and pseudo-time analysis

In GSE152805, in the OA group, the cell communication analysis was carried out with “CellChat” package (v 1.6.1) (42) between all cell types.

Later, in an attempt to look into how key cells differentiate and evolve during the course of development, a pseudo-time analysis was conducted. Specifically, in GSE152805, first, the key cells were regrouped (resolution = 0.4). Then, the “monocle” package (v 2.26.0) (43) was utilized to conduct a pseudo-time trajectory analysis of key cells. The purpose was to detect modifications in the developmental path of key cells along with the expression alterations.

### 2.12 Construction of OA rat model and sample collection

In total, six male Sprague–Dawley (SD) rats, aged 6–8 weeks, were obtained from Beijing Sibeifu Bio-Technology Co., Ltd. (Production License No. SCXK (Beijing) 2024-0001; Use License No. SYXK (Dian) K2022-0007). The rats were randomly assigned to two groups: the mock surgery group (three rats) and the osteoarthritis group (three rats). All rats were housed in a temperature-controlled environment with ad libitum access to food and water. Following a one-week acclimation period, the surgical procedures were carried out. In the OA group, after anesthesia, the rats were placed on the operating table, and their limbs were fixed. The area around the knee joint was wiped with povidone-iodine, and the surrounding hair was shaved clean. The area was then disinfected by spraying with 70% alcohol. Under aseptic conditions, the skin was incised longitudinally slightly medial to the patella to expose the surgical field of view. The joint capsule was opened through an incision on the medial side of the patellar ligament. The patella was pulled laterally and horizontally, and the joint capsule was further incised to fully expose the femoral condyle. The joint was flexed to expose the anterior cruciate ligament (ACL) and the anterior horn of the medial meniscus. The ACL was transected with a sterile surgical blade, and part of the meniscus was resected. During the surgical procedure, every effort was made to avoid damaging the articular cartilage. Subsequently, a drawer test was performed to check whether the ACL was transected. After the operation, the joint capsule and the skin were sutured layer by layer. Attention was paid to disinfecting each layer, and the rats were then returned to the breeding cage to be rewarmed and wait for recovery from anesthesia. In the control group, the joint capsule was opened using the above method, but neither the ACL transection nor the medial meniscectomy was performed. After disinfection, the incisions of the joint capsule and the skin were sutured layer by layer, and the rats were returned to the breeding cage to be rewarmed and wait for recovery from anesthesia. Subsequently, the body weights of the rats in both groups were measured and compared every week. The joint swelling degrees and joint activity scores of the rats in the two groups were compared on the experimental day, at 3 weeks, and at 6 weeks. At 6 weeks, cartilaginous tissue, blood, and right knee joint samples were gathered. Blood serum samples and samples of cartilaginous tissue were stored at −80 °C. The right knee joint samples were fixed in 4% paraformaldehyde for histological examination.

### 2.13 Hematoxylin and eosin and Masson staining

To compare the histopathological morphology of the right knee joint samples between the OA and control groups, hematoxylin and eosin (H&E) staining was performed, following several key steps. Initially, the right knee joint samples from three OA and three sham groups were taken and decalcified using an EDTA decalcifying solution. 4% paraformaldehyde was used to fix the decalcified samples, and the fixation time was set between 24 and 48 h. Fixed specimens were sequentially dehydrated with ethanol, transparentized with dimethylbenzene, paraffin-infiltrated, and embedded. The tissues were then cut into 5 micrometer-thick sections. For H&E staining, tissue sections were maintained at 64 °C for 1 h, dewaxed with xylene, and rehydrated by alcohol. Hematoxylin counterstaining was performed for 5 min, followed by eosin staining for 5–10 s. Samples were subjected to dehydration, clearing, and mounting, followed by whole-slide scanning analysis.

In order to further observe the degree of tissue fibrosis, Masson staining was performed. Following the decalcification treatment, the samples taken from the right knee joint were processed by embedding them in paraffin. Subsequently, these paraffin-embedded samples were cut into sections. The Masson staining process: dewax paraffin sections with dimethylbenzene, rehydrate with alcohol. Stain with Weigert iron hematoxylin for 5–10 min, differentiate in acidic ethanol for 5–15 s, wash. Reblue with Masson blue for 3–5 min, rinse, stain with ponceau fuchsin for 5–10 min. Wash with 2:1 distilled water-weak acid solution, treat with phosphomolybdic acid, wash again. Stain with aniline blue, rinse, then dehydrate, clear, and mount.

### 2.14 Immunohistochemical analysis

To detect protein expression levels of key genes, immunohistochemistry was conducted involving a series of key procedures. Initially, right knee joint samples from three OA and three sham rats were decalcified before fixation with 4% paraformaldehyde for 24–48 h. Fixed samples were alcohol-dehydrated, xylene-cleared, paraffin-infiltrated, and embedded, followed by cutting 3 μm-thick sections.

For the immunohistochemical (IHC) assay, tissue sections were incubated at 64 °C for 1 h, then xylene-dewaxed, alcohol-rehydrated, and subjected to antigen retrieval. Endogenous peroxidase activity was blocked using 3% H₂O₂, and samples were blocked with 5% BSA at 37 °C before treatment with 2% BSA-diluted primary antibodies (Supplementary Table S2). Next, 100 μL of signal enhancer was applied, followed by incubation with anti-mouse IgG conjugate. Diaminobenzidine (DAB) was used for color development, and hematoxylin counterstaining was performed for 5 min. After dehydration, clearing, and mounting, whole-slide scanning analysis was conducted.

### 2.15 RT-qPCR

With the aim of confirming the expression of key genes, RT-qPCR analysis was performed. Specifically, a total of six cartilaginous tissue samples (three OA and three sham) were utilized. Total RNA was extracted from the six samples using TRIzol (Ambion, Austin, United States). Reverse transcription to cDNA was carried out with the SureScript First-Strand cDNA Synthesis Kit (Servicebio, Wuhan, China), and the reverse transcription was carried out with S1000^™^ Thermal Cycler (Bio-Rad, United States). Primer sequences for the PCR were provided in Supplementary Table S3. RT-qPCR was conducted by the 2× Universal Blue SYBR Green qPCR Master Mix (Servicebio, Wuhan, China). GAPDH served as an internal reference gene. The relative quantification of key genes was measured by the 2^−ΔΔCT^ method (44). Finally, Graphpad Prism (v 5.0.0) (45) was applied to plot and work out the *p*-value (*p* < 0.05).

### 2.16 Western blot

The Western blot (WB) method was employed for measuring the protein expression levels of key genes. In this study, a total of six cartilaginous tissue samples (three OA and three sham) were utilized. Tissue lysates were prepared by adding 1 mL of RIPA buffer (Servicebio, G2002-30ML) supplemented with 10 μL of protease inhibitors (Proteintech, PR20032). Protein concentrations were determined using the BCA assay. For the assay, 80 μL of protein samples (3.8 to 6 μg/μL) were combined with an equal volume of 5× protein loading buffer and denatured by boiling for 10 min. After cooling to room temperature, the samples were stored at −80 °C. For SDS-PAGE, the spacer gel was prepared. Electrophoresis was conducted with 10 μL of the sample at 80 V for approximately 30 min. When the protein sample reached the separating gel interface, the voltage was raised to 120 V and electrophoresis was performed until the bromophenol blue dye reached the bottom of the gel, taking about 90 min. The gel was then removed, and proteins were transferred onto PVDF membranes at 4 °C using a current of 300 mA for 1 h. After transfer, the membranes were blocked with 5% BSA at 37 °C for 30 min. Subsequent to blocking, the membranes were placed in a solution containing primary antibodies directed against the key genes and then incubated overnight at a temperature of 4 °C (Supplementary Table S4). Following multiple washes using TBST, secondary antibodies, diluted 1:5,000 in 5% skim milk, were used to immerse the membranes, which were then incubated for 60 min. Subsequent to the previous steps, additional washes with TBST were carried out. Subsequently, the bands were made visible through the application of an ECL detection system. Images were captured with a gel imager.

### 2.17 Statistical analysis

The statistical analysis was done by means of R (v 4.2). Differences analysis between groups was executed via the Wilcoxon test (*p* < 0.05). Differences between PCR experimental categories were obtained through a *t*-test.

## 3 Results

### 3.1 Thirty-four candidate genes were engaged in complex signaling pathways and played various functional roles

**Figure 1:**
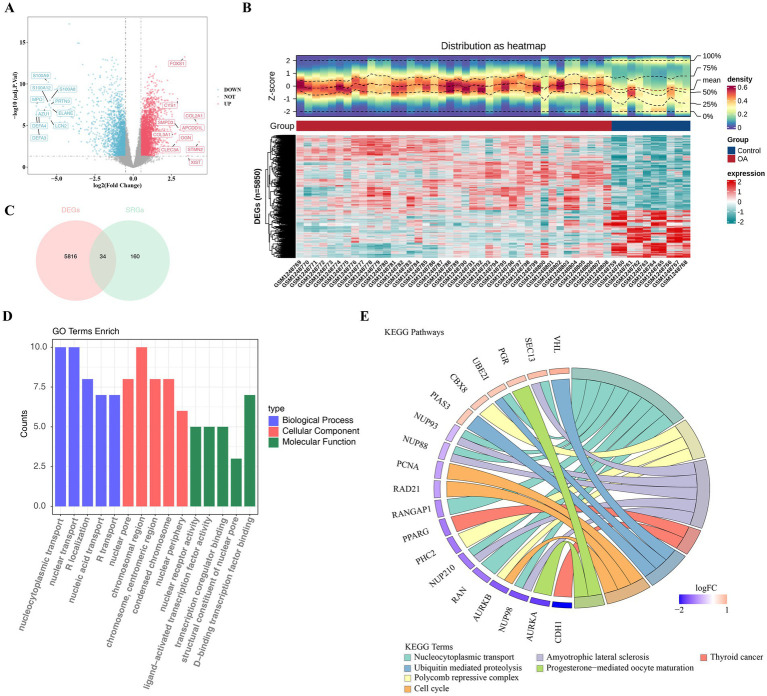
Identification of differentially expressed genes (DEGs) and enrichment pathways associated with osteoarthritis (OA) prognosis. **(A)** Volcano plot displaying DEGs between OA and control samples in the GSE51588 dataset. Genes with |log_2_FoldChange| >0.5 and adjusted *p*-value <0.05 are highlighted, with the top 10 upregulated and downregulated genes labeled based on fold change magnitude. **(B)** Heatmap illustrating the expression profiles of all DEGs across OA and control samples, showing distinct clustering of groups. **(C)** Venn diagram identifying 34 candidate genes by intersecting DEGs from GSE51588 with 194 SUMOylation-related genes (SRGs). **(D)** Bar plot of Gene Ontology (GO) enrichment analysis for candidate genes, showing the top five significantly enriched terms (adjusted *p*-value <0.05) in biological processes (e.g., nucleocytoplasmic transport), cellular components, and molecular functions. **(E)** Chord diagram of KEGG pathway enrichment, highlighting seven significantly enriched pathways (e.g., nucleocytoplasmic transport and amyotrophic lateral sclerosis) based on candidate genes.

Differential expression analysis identified 5,850 DEGs in GSE51588, with 3,577 showing high expression and 2,273 showing low expression in the OA group (Figures 1A,B). Critically, by intersecting these DEGs with a curated list of 194 SUMOylation-related genes (SRGs), we pinpointed 34 candidate genes that are both dysregulated in OA and functionally linked to SUMOylation (Figure 1C). This core gene set served as the foundation for all subsequent analyses, ensuring our focus remained on SUMOylation-associated pathways. Enrichment analysis revealed that 34 candidate genes were significantly associated with 277 GO terms, which included 215 biological processes (BPs), 41 cellular components (CCs), and 21 molecular functions (MFs). Subsequently, for GO, the enriched pathways were ranked from lowest to highest according to *p*-values, and the top five pathways were presented, notable GO terms included “nucleocytoplasmic transport” (BP), “chromosomal region” (CC), and “DNA-binding transcription factor binding” (MF) (Figure 1D and Supplementary Table S5). Additionally, there was a significant enrichment of candidate genes in seven KEGG pathways, including “nucleocytoplasmic transport,” “polycomb repressive complex,” “amyotrophic lateral sclerosis,” “thyroid cancer,” and so on (Figure 1E and Supplementary Table S6). All in all, these findings brought to light the important biological processes involved in OA and offered potential targets for further study.

### 3.2 NUP98 and TOP1 were chosen as key genes

**Figure 2:**
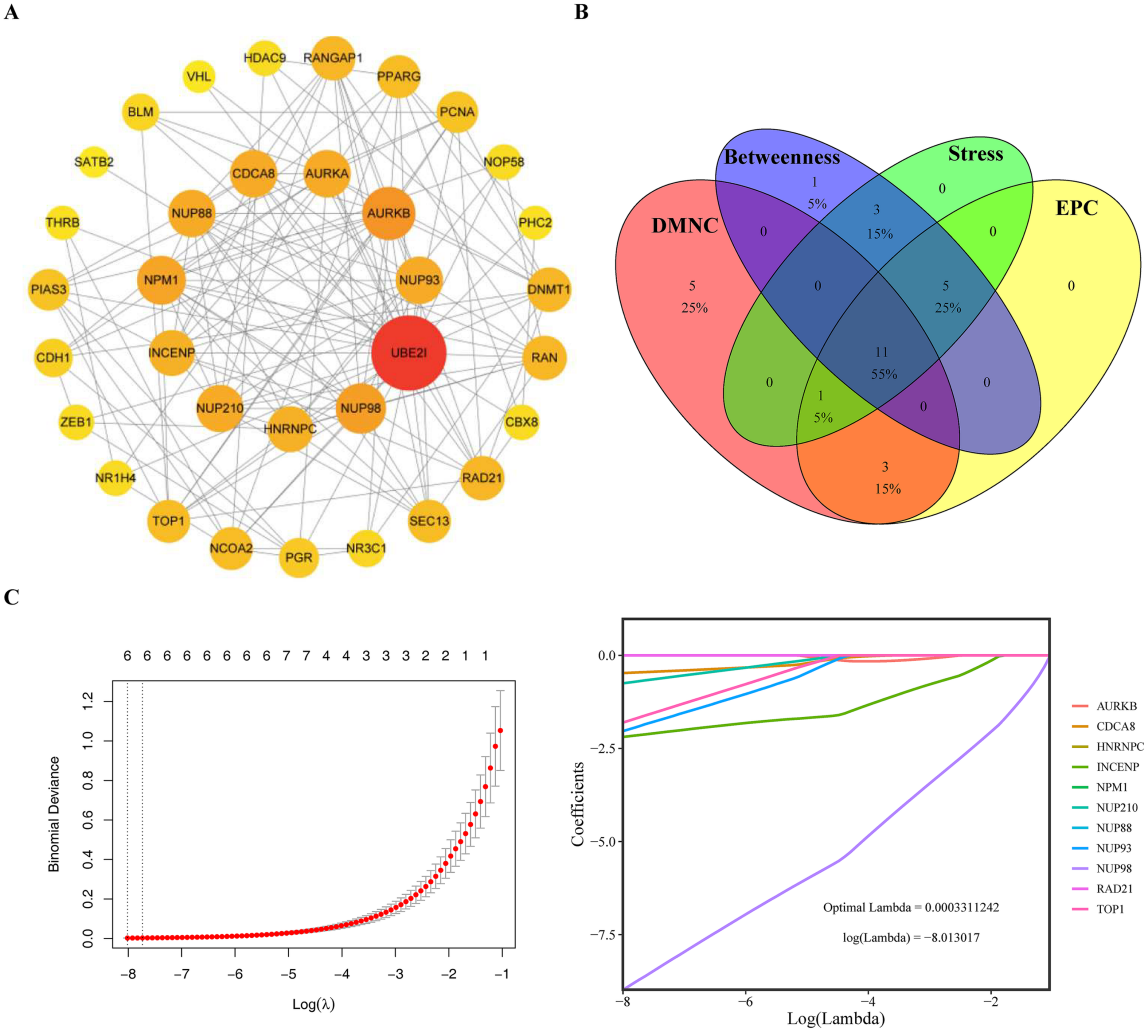
Screening of key genes **(A)**. PPI network of 34 candidate genes constructed using STRING database (interaction score ≥0.4), depicting 150 interactions among proteins. The network was visualized with Cytoscape software **(B)**. Identification of 11 hub genes from the PPI network using four centrality algorithms (DMNC, EPC, Betweenness, and Stress), with the top 20 genes from each algorithm intersected **(C)**. LASSO regression analysis performed on the GSE51588 dataset to select feature genes. The plot shows the coefficient distribution of genes across lambda values, with 10-fold cross-validation used to determine the optimal lambda (dotted vertical line). Six genes with non-zero coefficients were retained as feature genes.

A PPI network including 150 reciprocal relationships of 34 proteins was created (Figure 2A). Using multiple centrality algorithms, we identified 11 high-confidence hub genes within this network (Figure 2B). To enhance the robustness of our selection and mitigate overfitting, we subsequently applied LASSO regression analysis. This machine learning approach narrowed the list to six feature genes with the strongest predictive power for OA (Figure 2C).

**Figure 3:**
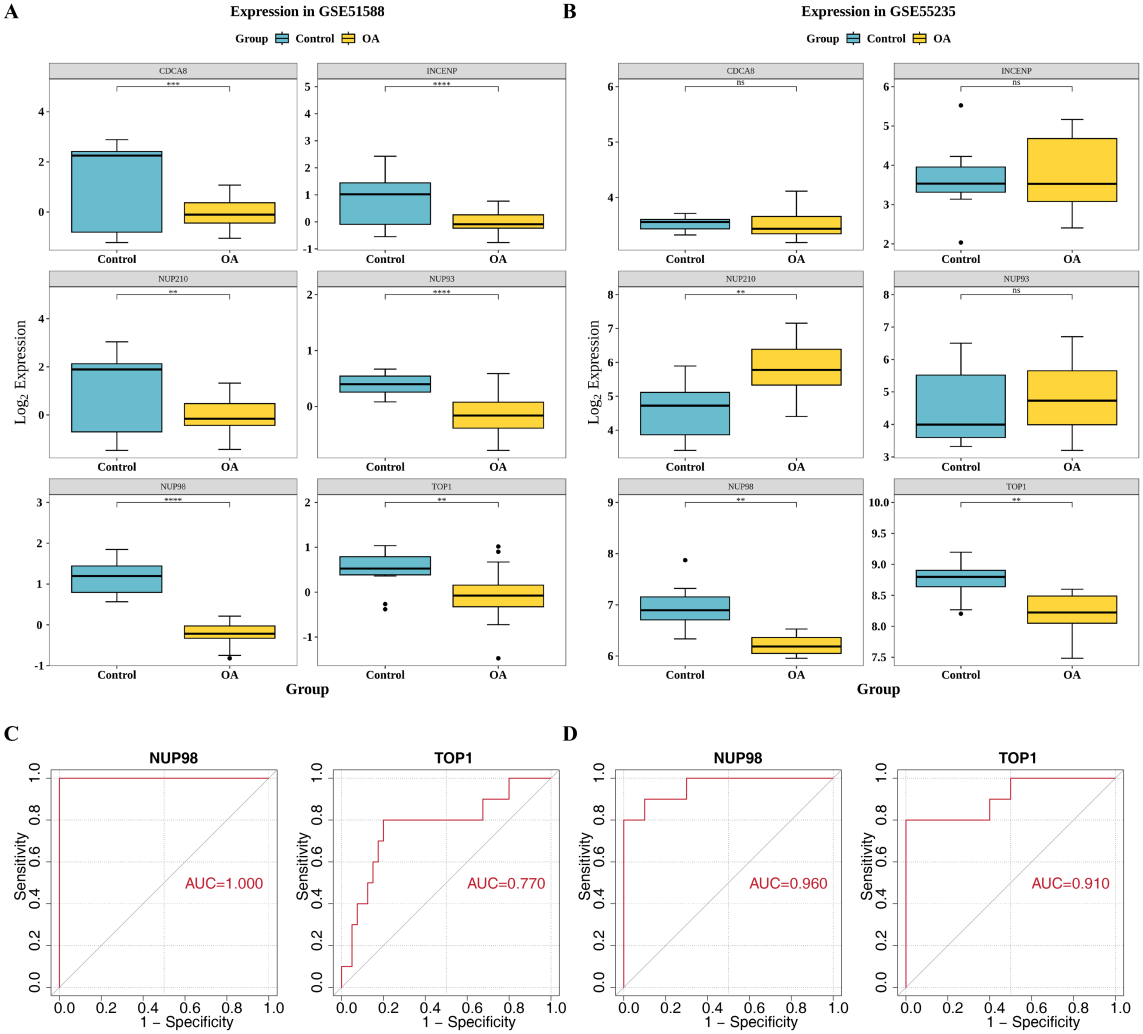
Validation of candidate key genes and assessment of their diagnostic value. **(A,B)** Box plots showing the expression levels of six feature genes (identified by LASSO regression) in the **(A)** GSE51588 (training set) and **(B)** GSE55235 (validation set) datasets. Wilcoxon test was used to compare OA and control groups (^*^p < 0.05). NUP98 and TOP1 showed consistent downregulation in both datasets. **(C,D)** Receiver operating characteristic (ROC) curves evaluating the diagnostic potency of NUP98 and TOP1 in **(C)** GSE51588 and **(D)** GSE55235. The area under the curve (AUC) values exceeded 0.7 for both genes, indicating strong discriminatory ability.

Subsequently, the expression analysis results of six feature genes indicated that two candidate key genes (NUP98 and TOP1) exhibited significant differential expression levels between disease and normal groups from GSE51588 and GSE55235 (*p* < 0.05) and had the same expression trend in both datasets (Figures 3A,B). Meanwhile, the AUC values of both two genes were >0.7 in both datasets, indicating that these genes demonstrated strong capability in discriminating OA and control samples (Figures 3C,D). Therefore, NUP98 and TOP1 were selected as the key SUMOylation-related genes driving OA progression.

To address potential tissue-specific concerns, we performed an independent validation using cartilage transcriptomic datasets. In GSE114007 (20 OA vs. 18 normal), NUP98 showed significant downregulation (log_2_FC = −0.67, adj. *p* = 5.48 × 10^−6^), while TOP1 exhibited a non-significant decreasing trend (log_2_FC = −0.30, adj. *p* = 0.25). ROC analysis confirmed the diagnostic value of both genes (AUC = 0.738). GO enrichment revealed significant pathways like ossification and extracellular matrix organization, with weak trends in nucleocytoplasmic transport and translation, aligning with SUMOylation-related functions. Randomized control analyses demonstrated that the cross-tissue expression consistency of NUP98 and TOP1 significantly exceeded 99% of random gene sets (*p* < 0.01), indicating it is not a tissue-intrinsic feature (Supplementary Figure S1A). For the 11,790 genes common to all three joint tissues (cartilage, subchondral bone, synovium), we identified 15 target genes co-expressed with NUP98 or TOP1. Specifically, 13 of 15 target genes (86.7%) showed significant co-expression with NUP98 across all tissues, while 10 of 15 (66.7%) co-expressed with TOP1. For NUP98, 86.7% (13/15) of its target genes exhibited significant correlations (|*r*| > 0.3, *p* < 0.05) in all tissues. This conservation ratio vastly surpassed the 99th percentile of 1,000 random gene sets (6.7%), with an empirical *p*-value <0.001 (exceeding all random sets). As shown in Supplementary Figure S1B, both NUP98 (86.7%) and TOP1 (66.7%) far exceeded the random expectation (6.7%), with significance surpassing 99% of random sets (*p* < 0.001), thereby eliminating coincidental overlap as a plausible explanation. Thus, two genes (NUP98 and TOP1) were deemed as key genes.

### 3.3 Integrative analysis of consistent NUP98 and TOP1 dysregulation in osteoarthritis across joint tissues

To directly address whether NUP98 and TOP1 exhibit consistent dysregulation across distinct joint tissues, we integrated four independent datasets (GSE117999, GSE82107) into a unified cohort (107 samples; 54 controls/53 OA). Robust batch effect correction was applied, as evidenced by the improved distribution of gene expression across samples (Supplementary Figure S2A) and the clear separation of OA and control groups in the principal component analysis (PCA) plot post-correction (Supplementary Figure S2B). In this integrated cohort, both NUP98 and TOP1 demonstrated significant differential expression in OA compared to controls (Wilcoxon test, *p* < 0.05; Supplementary Figure S2C), with consistent directionality (downregulation). Furthermore, a significant albeit weak positive correlation was observed between the expression levels of NUP98 and TOP1 across all samples (Spearman’s *ρ* = 0.306, *p* = 0.00134; Supplementary Figure S2D), suggesting their potential co-involvement in a shared, though not strictly coordinated, molecular network in OA pathogenesis.

### 3.4 The nomogram model demonstrated a high predictive capacity for OA

**Figure 4:**
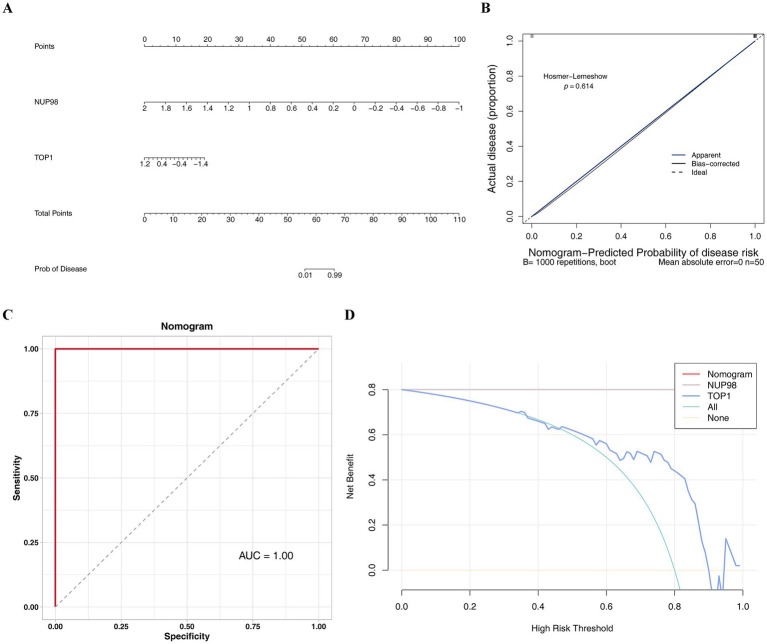
Construction and validation of a diagnostic nomogram model for OA. **(A)** Nomogram developed based on key genes (NUP98 and TOP1) in the GSE51588 dataset. The total score sums points assigned to each gene’s expression level, with higher scores indicating increased OA risk. **(B)** Calibration curve plotting predicted probabilities against observed frequencies. A slope close to 1 indicates high consistency between model predictions and actual outcomes. **(C)** ROC curve of the nomogram model, with an AUC of 1.00, demonstrating perfect discrimination between OA and control samples. **(D)** Decision curve analysis (DCA) evaluating the clinical utility of the nomogram. The model’s net benefit (blue curve) exceeds that of all key genes individually and the treat-all or treat-none strategies, confirming robust diagnostic performance.

A predictive nomogram model was developed where a higher total score elevated the risk of developing OA (Figure 4A). Calibration curves with slopes approaching one demonstrated strong consistency between the probability predicted by the model and the observed incidence rate (Figure 4B). The AUC value of the ROC curve was 1.00 demonstrating that the higher efficacy of the model to distinguishing disease from nondisease (Figure 4C). The net benefit from DCA analysis exceeded 0, and the nomogram model’s net benefit surpassed that of all key genes (the NUP98 curve covered the model curve), further confirming that the model possessed robust diagnostic prediction capacity (Figure 4D). In general, this analysis further probed into the diagnostic predictive capabilities of key genes in OA.

### 3.5 Key genes were enriched in multiple pathways, which might influence the development of OA

**Figure 5:**
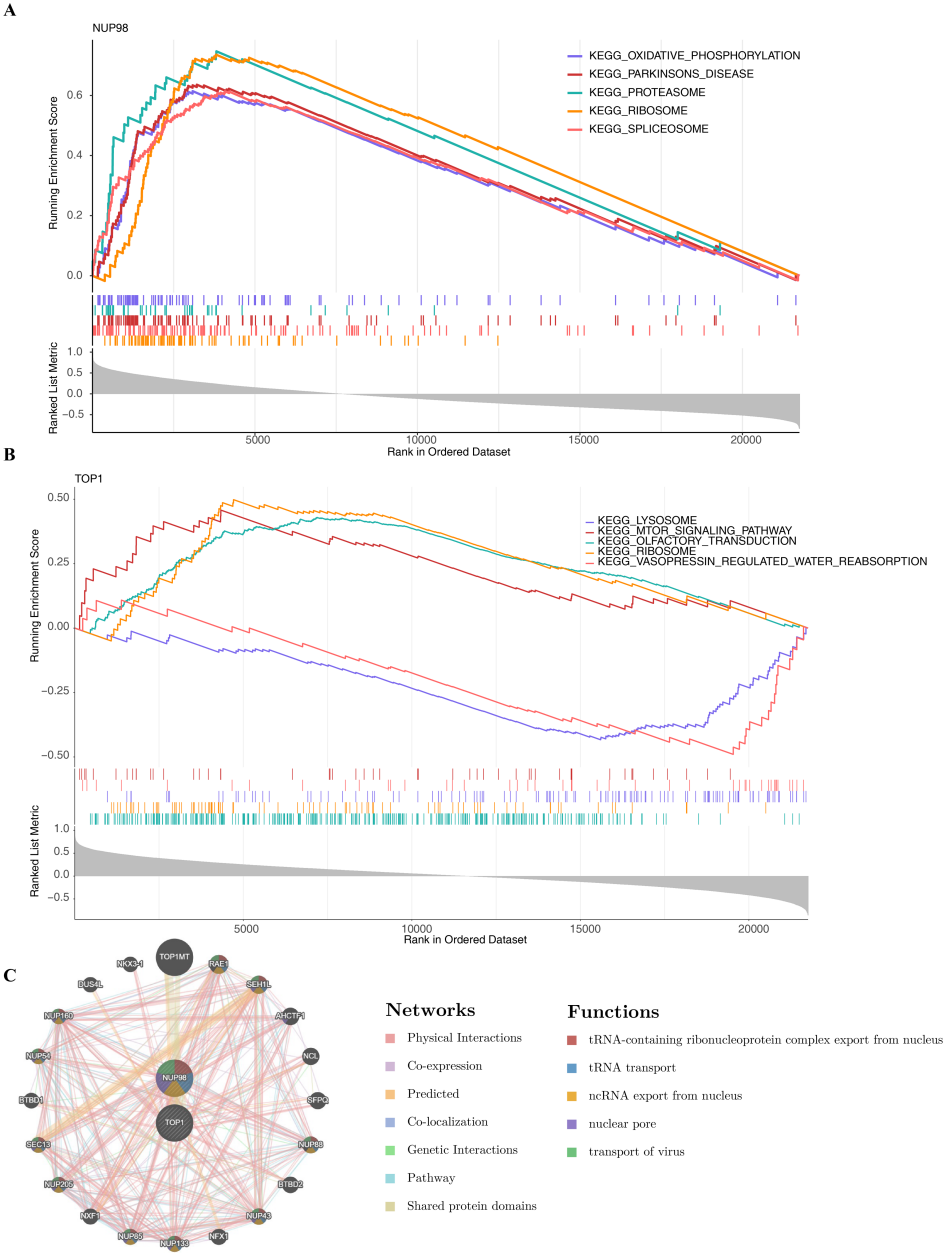
Functional enrichment and interaction network analysis of key genes NUP98 and TOP1. **(A,B)** Gene set enrichment analysis (GSEA) results showing the four pathways commonly enriched for both NUP98 and TOP1: ribosome, olfactory transduction, nucleotide excision repair, and endocytosis. The plots display normalized enrichment scores (NES) and false discovery rate (FDR) values. **(C)** Functional interaction network generated using GeneMANIA, depicting 20 genes functionally linked to NUP98 and TOP1. Edges represent interaction types (e.g., co-expression, physical interactions), with key mechanisms including tRNA transport and nuclear pore organization.

In order to elucidate the potential roles of NUP98 and TOP1 in the pathogenesis of osteoarthritis (OA), we conducted a gene set enrichment analysis (GSEA). As demonstrated by the GSEA results, NUP98 was significantly enriched in 74 pathways (Supplementary Table S7), and TOP1 was closely related to seven biological pathways (Supplementary Table S8). While NUP98 and TOP1 each connect to numerous pathways, the two key genes were significantly co-enriched in four pathways, including “ribosome,” “olfactory transduction,” “nucleotide excision repair,” and “endocytosis” (Figures 5A,B).

Through GeneMANIA analysis, 20 genes related to the function of the key genes were identified. These genes mainly interact with key genes through five different mechanisms, including “tRNA transport,” “nuclear pore,” and “transport of virus,” and so on (Figure 5C). These analyses offered insights into their potential involvement in the progression of OA.

### 3.6 GSEA reveals convergent enrichment of NUP98 and TOP1 in mRNA processing pathway across tissues

GSEA demonstrated that both genes were significantly and concordantly enriched in the mRNA processing pathway (false discovery rate, FDR <0.001) in all examined tissues (Supplementary Figures S3A,B). While both genes showed significant enrichment, TOP1 exhibited a stronger association with this pathway (normalized enrichment score, NES = 2.43) compared to NUP98 (NES = 1.98). The enrichment plot for TOP1 also showed a more concentrated leading-edge distribution, suggesting a more focused core of genes driving the enrichment. This convergent enrichment provides compelling evidence that NUP98 and TOP1 coregulate a fundamental post-transcriptional mechanism-mRNA processing-across the central joint tissues affected by OA.

### 3.7 Key genes showed correlation with immune cells

**Figure 6:**
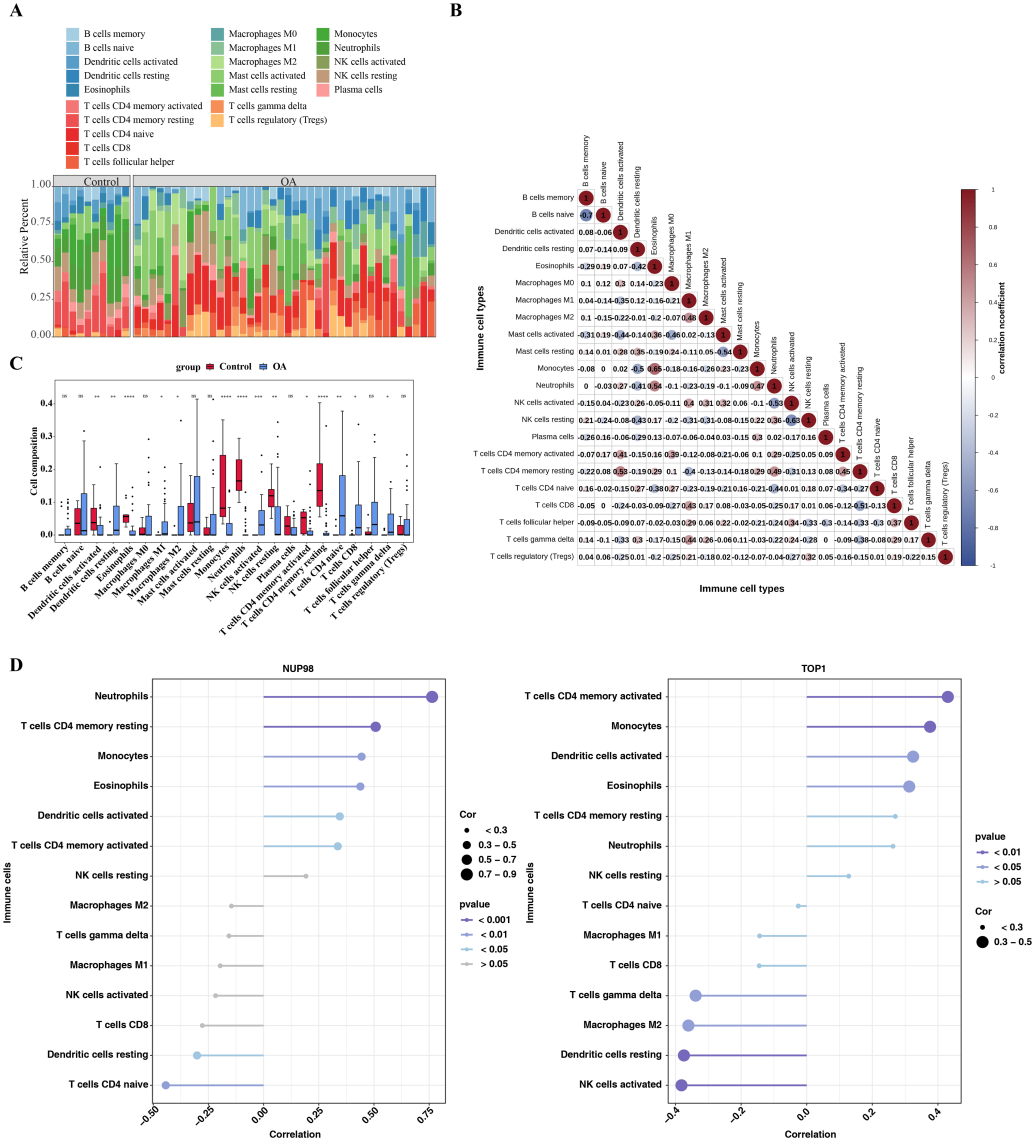
Immune infiltration landscape and correlation with key genes in OA. **(A)** Stacked bar chart showing the proportional distribution of 22 immune cell types in OA and control samples, calculated using the CIBERSORT algorithm. **(B)** Correlation network of immune cell types based on Spearman correlation coefficients (|cor| >0.3 and *p* < 0.05). Red edges indicate positive correlations, blue edges negative correlations. **(C)** Bar plot displaying 14 immune cell types with significant abundance differences between OA and control groups (Wilcoxon test, *p* < 0.05), including monocytes and neutrophils. **(D)** Heatmap of Spearman correlations between key genes (NUP98 and TOP1) and immune cells. Significant correlations (|cor| >0.3, *p* < 0.05) are highlighted, with color intensity indicating correlation strength.

From the stacked chart of cell proportions, one could see the proportions of 22 types of immune cells in the OA as well as the control samples (Figure 6A). Moreover, naive B cells had the strongest significant negative correlation with memory B cells (cor = −0.70, *p* = 2.30 × 10^−3^), and monocytes had the strongest significant positive correlation with eosinophils (cor = 0.65, *p* = 6.19 × 10^−5^) (Figure 6B and Supplementary Table S9). Revealed significant associations between key genes and specific immune cell subsets, for example, a total of 14 immune cell types had notable differences between the OA and control groups, including monocytes, neutrophils, and so on (*p* < 0.05) (Figure 6C). Meanwhile, NUP98 had the strongest significant positive correlation with neutrophils (cor = 0.76 and *p* < 0.001) and had the strongest significant negative correlation with native CD4 T cells (cor = −0.44 and *p* < 0.01), respectively. TOP1 had the strongest significant positive correlation with activated CD4 memory T cells (cor = 0.43 and *p* < 0.01) and had the strongest significant negative correlation with activated natural killer (NK) cells (cor = −0.38 and *p* < 0.01), respectively (Figure 6D and Supplementary Table S10). These associations imply that NUP98 and TOP1 may act directly in chondrocytes and, through regulating immune cell infiltration in the joint, indirectly participate in the inflammatory progression of OA.

### 3.8 Key genes-associated regulatory molecules and drugs were predicted

**Figure 7:**
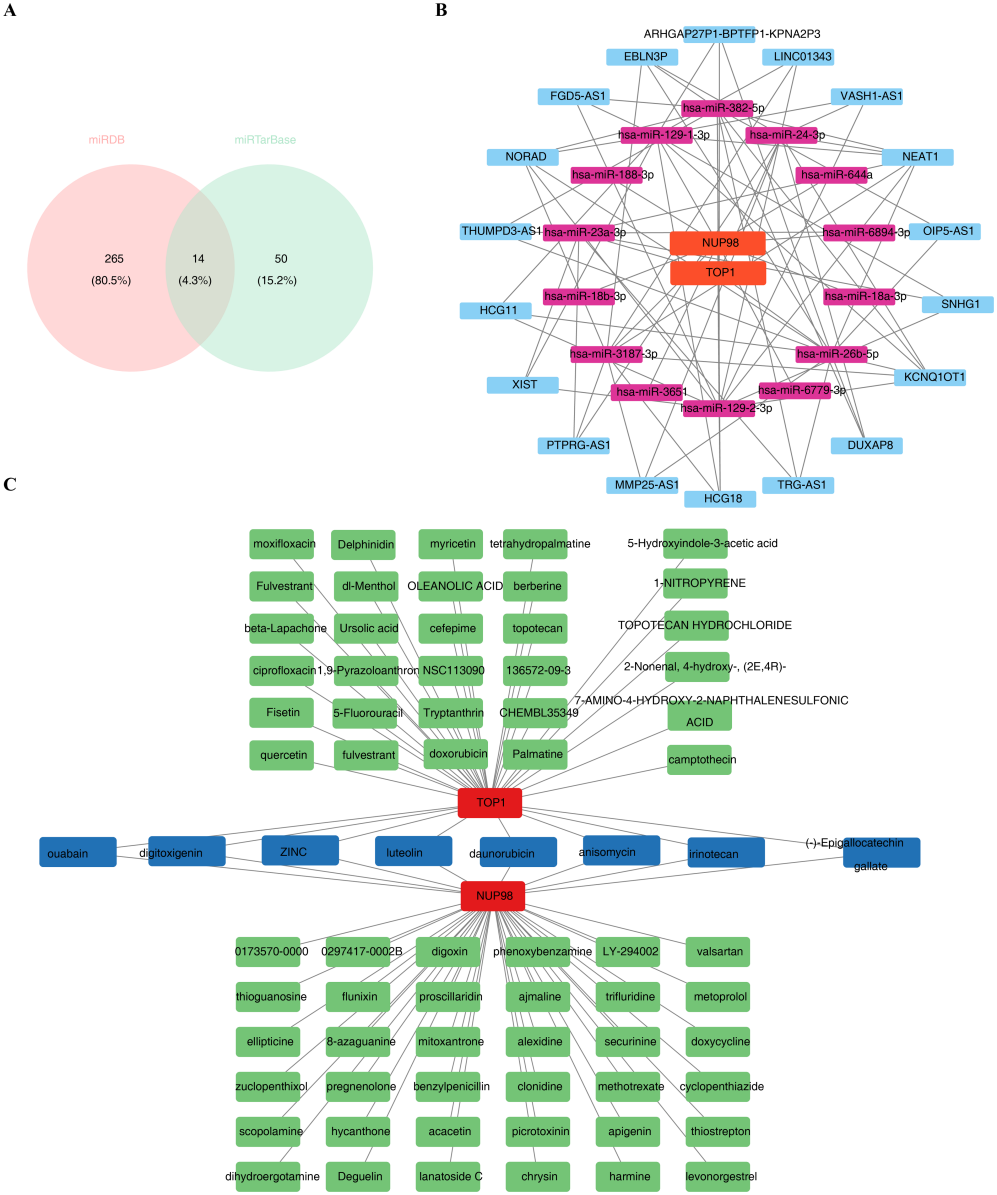
Regulatory networks and potential therapeutic agents targeting key genes in OA. **(A)** Venn diagram illustrating 14 shared miRNAs predicted to interact with *NUP98* and *TOP1*, identified from miRTarBase and miRDB databases. **(B)** Competing endogenous RNA (ceRNA) network comprising 14 miRNAs, 18 lncRNAs (e.g., *NEAT1*), and two key genes, constructed using Cytoscape. Edges represent regulatory interactions (e.g., miRNA-lncRNA binding). **(C)** Drug-gene interaction network highlighting 44 drugs targeting *NUP98* and 38 drugs targeting *TOP1*, predicted using the Enrichr database. Eight drugs (e.g., luteolin) are associated with both genes.

There were 14 shared miRNAs that were shared by two databases. Of these, 10 miRNAs (such as hsa-miR-18a-3p) were associated with NUP98 and four miRNAs (hsa-miR-23a-3p, hsa-miR-26b-5p, hsa-miR-382-5p and hsa-miR-24-3p) were associated with TOP1 (Supplementary Table S11 and Figure 7A). Based on these 14 shared miRNAs, a total of 18 lncRNAs were identified (Supplementary Table S12). The lncRNA-miRNA-key gene regulatory network included 14 miRNAs, 18 lncRNAs, and two key genes. Among them, the lncRNA (NEAT1) targeted seven miRNAs (hsa-miR-129-1-3p, hsa-miR-129-2-3p, hsa-miR-23a-3p, hsa-miR-24-3p, hsa-miR-26b-5p, hsa-miR-3187-3p, and hsa-miR-382-5p) (Figure 7B). To sum up, these findings underlined the regulatory networks related to miRNAs and lncRNAs that might have an effect on OA.

Subsequently, we built a drug-key gene network encompassing 44 drugs targeting NUP98, and 38 drugs targeting TOP1, among which eight drugs (digitoxigenin, anisomycin, luteolin, irinotecan, ZINC, (−)-epigallocatechin gallate, daunorubicin, and ouabain) were simultaneously associated with both two key genes (Figure 7C). Overall, these findings offered a new path for choosing therapeutic drugs for OA.

### 3.9 There were nine cell types annotated

**Figure 8:**
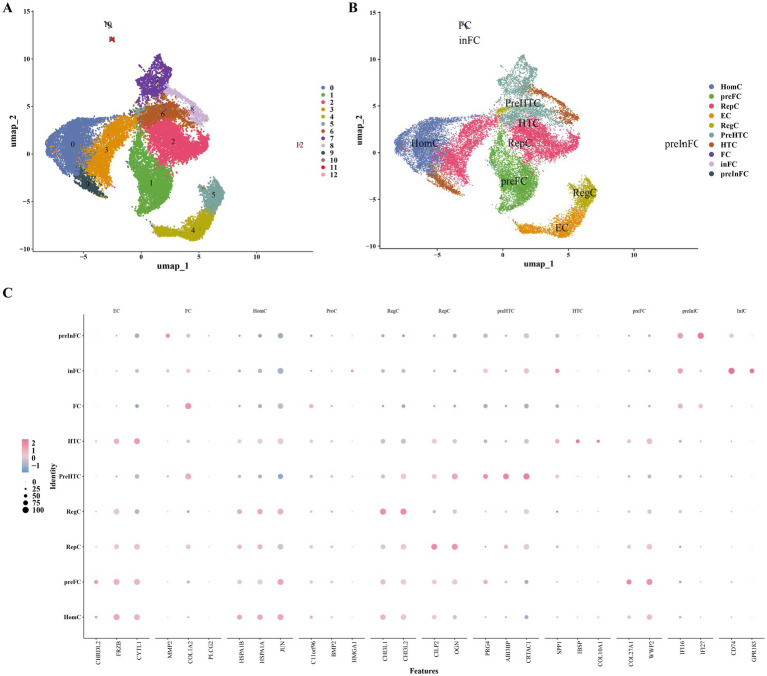
Single-cell RNA sequencing analysis of chondrocyte heterogeneity in osteoarthritic samples. **(A)** UMAP plot showing 13 distinct cell clusters identified after quality control and unsupervised clustering (resolution = 0.4). **(B)** Annotation of the 13 clusters into nine chondrocyte subtypes based on marker genes from literature, including homeostatic chondrocytes (HomCs) and fibrocartilage chondrocytes (FCs). **(C)** Bubble plot depicting the expression of marker genes across chondrocyte subtypes, with dot size and color indicating expression level and percentage of cells expressing the gene.

After screening the single-cell sequencing data from six OA samples, altogether 21,236 genes and 25,321 cells were identified. The results before and after quality control (QC) were presented in Supplementary Figures S4A,B. A total of 2,000 HVGs were selected for subsequent analysis; the top 10 genes that showed the most significant changes in intercellular expression were marked (Supplementary Figure S4C). The top 30 PCs were picked out for later analysis (Supplementary Figures S4D,E). In total, 13 different cell clusters were identified (Figure 8A). Moreover, the 13 clusters were annotated to nine cell types, including homeostatic chondrocytes (HomC), pre-fibrocartilage chondrocytes (preFC), reparative chondrocytes (RepC), regulatory chondrocytes (RegC), pre-hypertrophic chondrocytes (preHTC), hypertrophic chondrocytes (HTC), fibrocartilage chondrocytes (FC), inflammatory fibrocartilage chondrocytes (inFC), and pre-inflammatory fibrocartilage chondrocytes (preInFC) (Figure 8B). It was demonstrated by the bubble plot that high specificity was exhibited by these marker genes (Figure 8C).

### 3.10 HomCs were selected as the key cells and had some communication with some other annotated cell types

**Figure 9:**
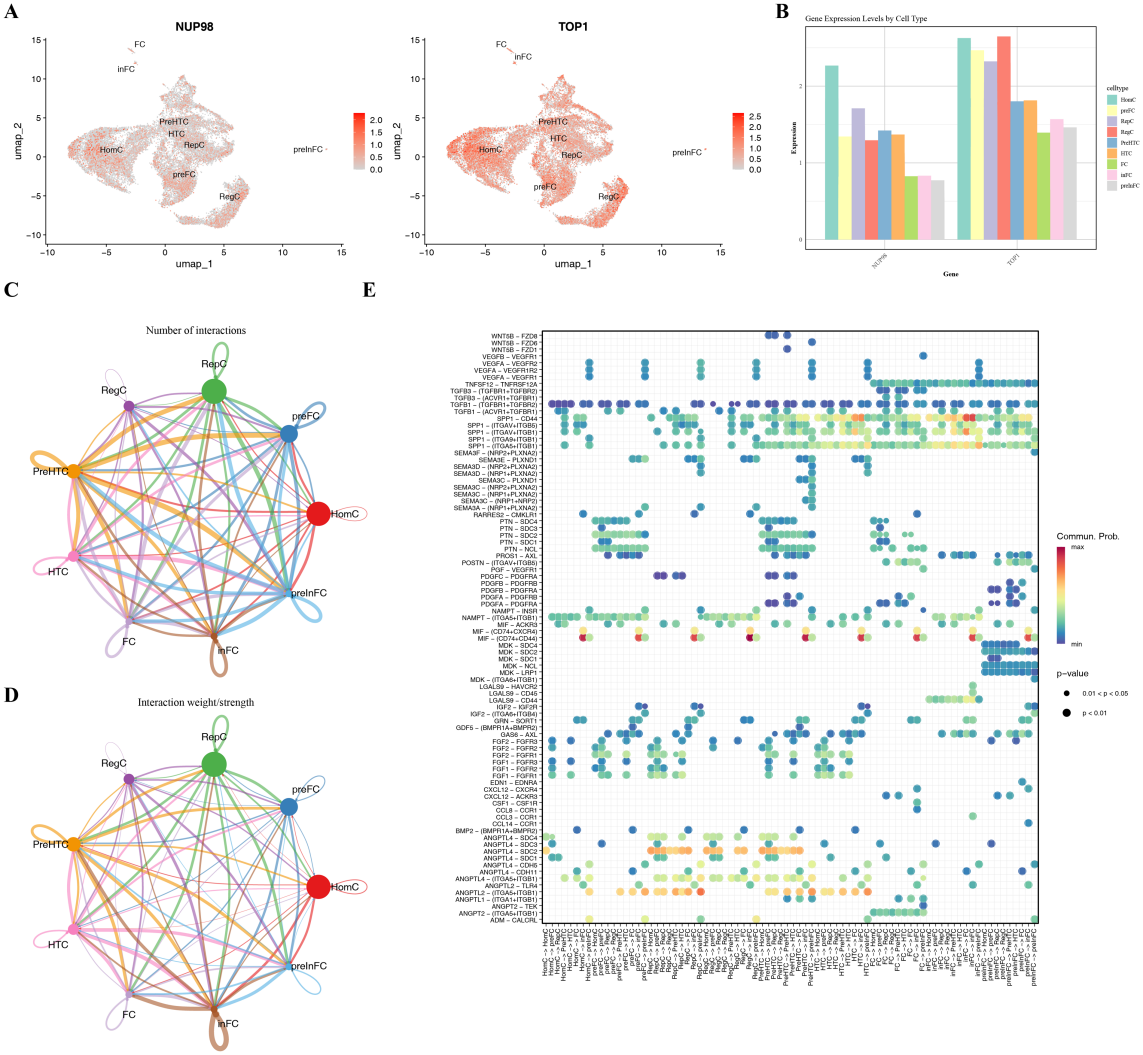
Single-cell expression and intercellular communication of key genes in chondrocyte subtypes. **(A)** Violin plots showing the expression distribution of *TOP1* and *NUP98* across all cells in the OA samples. **(B)** Bar plot of average expression levels of *TOP1* and *NUP98* across the nine annotated chondrocyte subtypes, with HomCs showing the highest expression. **(C)** Intercellular communication network inferred using CellChat, depicting the number of interactions between HomCs and other subtypes. **(D)** Interaction weight analysis revealing stronger communication strength between HomCs and preFCs/preHTCs. **(E)** Specific ligand-receptor pairs [e.g., MIF-(CD74^+^ CD44)] mediating communication between inflammatory fibrocartilage chondrocytes (inFC) and HomCs.

Having identified NUP98 and TOP1 as key genes at the bulk tissue level, we next sought to pinpoint the specific cellular context of their action within the complex articular microenvironment using scRNA-seq data. Interrogating the expression distribution of NUP98 and TOP1 across all chondrocyte subtypes revealed that homeostatic chondrocytes (HomCs) displayed the highest average expression levels for both genes (Figures 9A,B). This striking specificity suggests that the dysregulation of SUMOylation pathways mediated by NUP98 and TOP1 predominantly occurs within HomCs, positioning this cell population as a critical nexus for OA pathology. Consequently, we designated HomCs as the key cell type for subsequent deeper analysis. In addition, in the intercellular communication networks, HomCs had communications with the other annotated cell types, among them the interaction numbers between HomCs and preFCs, as well HomCs and preHTCs HomCs and preInFCs, were relatively higher than the interaction numbers between HomCs and others (Figure 9C). From the perspective of interaction strengths, the interactions between HomCs and InFCs, as well as HomCs and preInFCs, were stronger than those between HomCs and the other annotated cells (Figure 9D). The results displayed the pairing of receptors and ligand in all cell types. For instance, in the OA group, the communication between InFC and HomCs, as well as InFC and HomCs, was mediated by MIF-(CD74^+^ CD44) (Figure 9E). Overall, there was a possibility that key cells influenced OA by way of intercellular interactions.

### 3.11 The expression levels of key genes changed over time with the differentiation of HomCs

**Figure 10:**
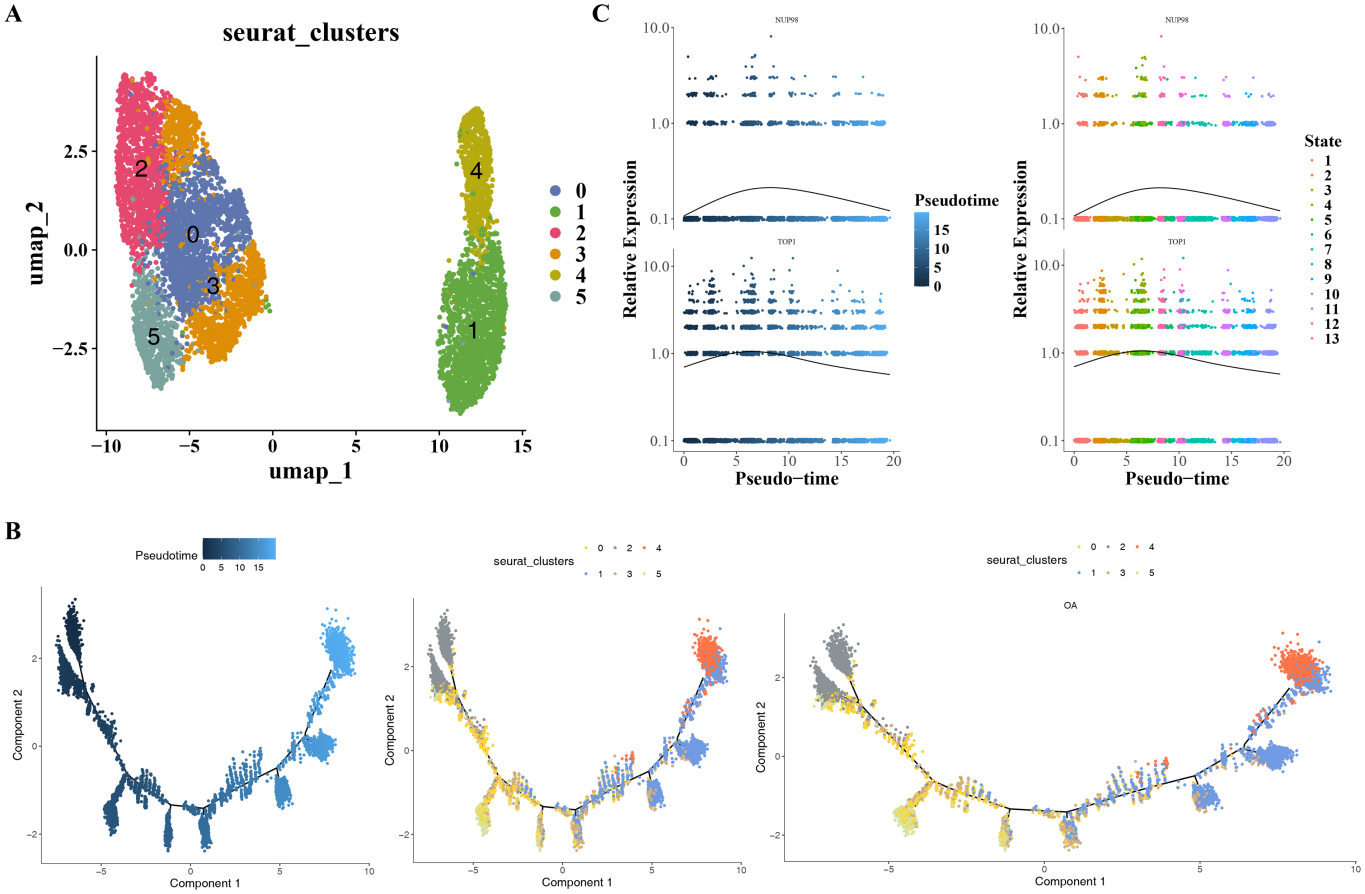
Subclustering and differentiation trajectory of homeostatic chondrocytes (HomCs). **(A)** UMAP projection of HomCs subclustered into seven distinct subtypes in the GSE152805 dataset. **(B)** Pseudotime trajectory analysis of HomCs, revealing three differentiation states and seven subtypes. **(C)** Expression dynamics of key genes *NUP98* and *TOP1* along chondrocyte differentiation.

In GSE152805, secondary clustering analysis based on HomCs was performed. Firstly, it reclassified the HomCs into seven distinct subtypes (resolution = 0.4) (Figure 10A). Following this, by observing the differentiation trajectory of HomCs, it was found that cells differentiated from the bottom left up and then back to the right over time. HomCs had a total of three different differentiation states and seven subtypes, among them, state 1 was the earliest differentiated type (Figure 10B). During the differentiation of HomCs, the expression level of both NUP98 and TOP1 first increased and then remained stable (Figure 10C). Overall, the distribution and expression of key genes were investigated at the cellular level. This approach effectively established a connection between genes and cells, thereby indicating a novel direction for subsequent research on the underlying mechanisms.

### 3.12 Collagen fibrosis was significantly more severe in the OA-group rats than in the sham-group rats

**Figure 11:**
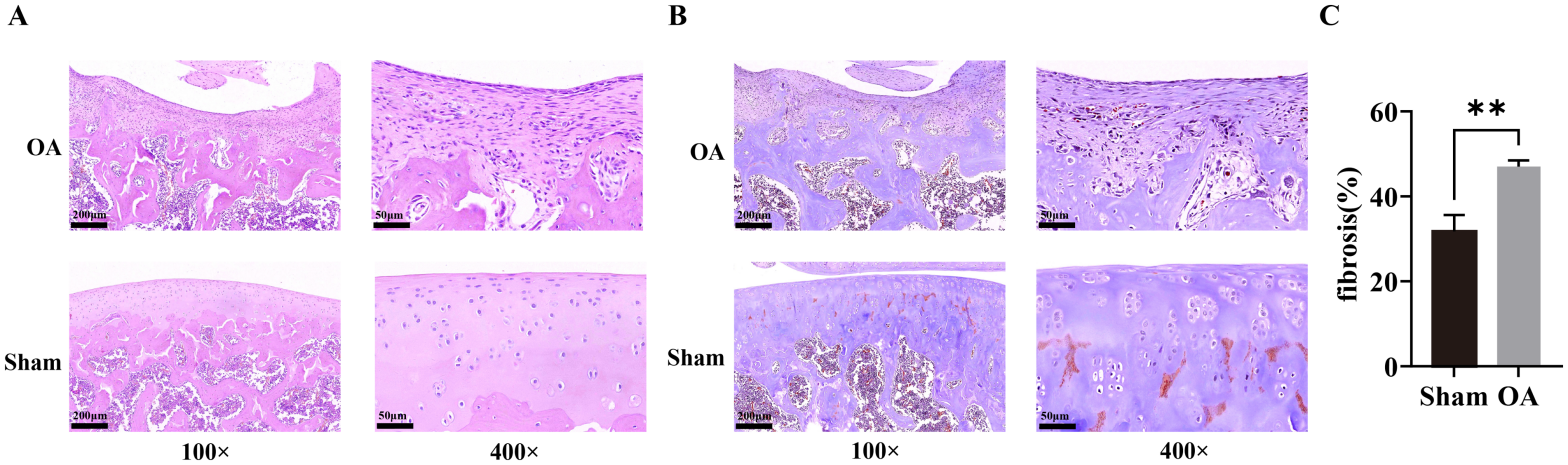
Histopathological evaluation of joint damage and collagen fibrosis in a rat OA model. **(A)** Representative hematoxylin and eosin (H&E)-stained images of knee joint sections from sham and OA groups. The OA group shows severe surface wear compared to the smooth sham group. **(B)** Masson’s trichrome staining highlighting collagen deposition (blue) in joint tissues. **(C)** Quantification of collagen fiber content using image analysis. Data are presented as mean ± SD; ^**^p < 0.01 by *t*-test, indicating significant fibrosis in the OA group.

In the OA animal model, the body weights of rats in the OA and sham groups increased gradually with time. The overall body weight of the OA group was lower, and there was a relatively large difference between the two groups from the 1st week to the 3rd week (Supplementary Figure S5A). Based on the joint swelling degree, it was shown that the bilateral joints of the rats in the OA group remained swollen continuously after the operation. The degree of swelling was more obvious from 0 to 3 weeks post-operation, and the swelling rate slowed down from 3 to 6 weeks (Supplementary Figure S5B). And, the joint activity score of the OA group continued to rise after the establishment of the model (Supplementary Figure S5C). Subsequently, after HE staining, it was found that the knee joint surface of the rats in the sham group was smooth and flat, while the joint surface of the rats in the OA group was severely worn (Figure 11A). Finally, Masson staining was used to detect the changes in collagen fibers in the tissues. The findings indicated that, in contrast to the sham group, the extent of collagen fibrosis in the OA group was significantly greater (*p* < 0.05, Figures 11B,C). The association between OA and collagen fibrosis was further affirmed by this finding.

### 3.13 The gene and protein expression of key genes was verified in the *in vitro* experiments

**Figure 12:**
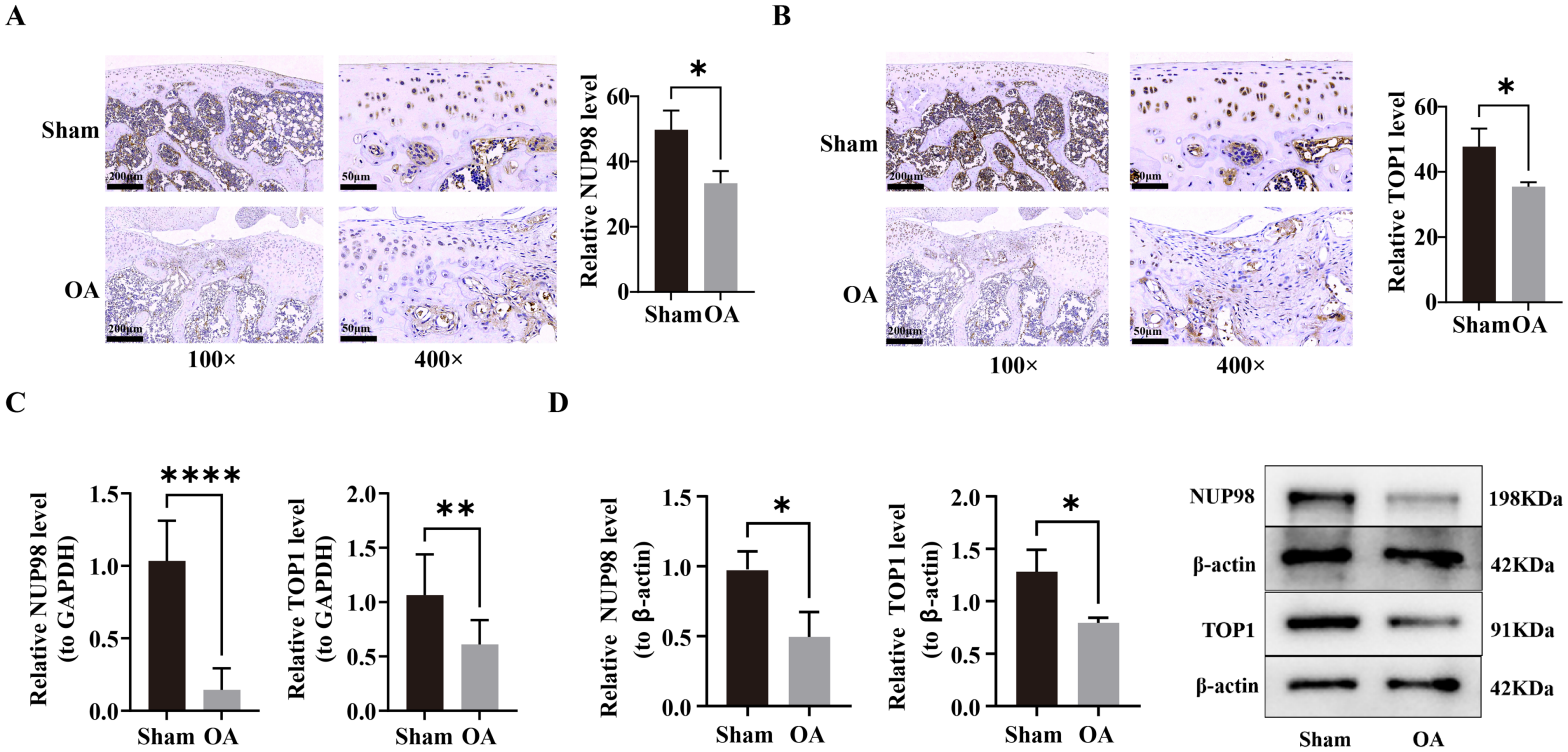
Validation of key gene expression in OA articular tissues. **(A,B)** Immunohistochemistry (IHC) staining and quantification of **(A)** NUP98 and **(B)** TOP1 protein expression in joint tissues. Signal intensity was lower in the OA group (^*^p < 0.05 by Wilcoxon test). **(C)** RT-qPCR analysis of NUP98 and TOP1 mRNA expression, normalized to GAPDH. The OA group showed significant downregulation (*p* < 0.01 for TOP1; ^**^p < 0.0001 for NUP98 by *t*-test). **(D)** Western blot analysis confirming decreased protein levels of NUP98 (198 kDa) and TOP1 (91 kDa) in the OA group, with β-actin (42 kDa) as loading control (^*^p < 0.05).

To ultimately validate the bioinformatics-based findings, we conducted in vitro experiments. IHC analysis revealed that the protein expression levels corresponding to two key genes, NUP98 and TOP1, were significantly reduced in the OA group compared with the sham group (*p* < 0.05, Figures 12A,B). Similarly, RT-qPCR confirmed a marked decrease in the mRNA expression levels of NUP98 and TOP1 in the OA group (*p* < 0.05, Figure 12C). Further validation by Western blot analysis corroborated these findings, confirming that the protein expression of NUP98 and TOP1 was significantly lower in the OA group (*p* < 0.05, Figure 12D). The consistency across these three methodologies highlighted the robustness of the findings. High consistency across platform-based validations not only confirms bioinformatics prediction reliability, but also further supports NUP98 and TOP1 as key genes in OA.

## 4 Discussion

OA is a complex degenerative joint disease with a pathogenesis involving multiple factors. Given the whole-joint pathological characteristics of OA, cross-tissue analysis of cartilage, subchondral bone and synovium has become a reliable strategy for exploring OA molecular mechanisms, and several studies have successfully identified OA biomarkers and regulatory signatures by integrating multi-tissue transcriptomic data with machine learning algorithms (46, 47), which provides a solid methodological precedent for our cross-tissue analytical framework in this study. In recent years, the role of protein SUMOylation in maintaining cartilage homeostasis and pathological progression of OA has increasingly been recognized (48–52). This study aims to systematically identify key molecules and cells associated with SUMOylation by integrating OA transcriptomic (GSE51588, GSE55235) and single-cell sequencing (GSE152805) datasets, and to explore their underlying mechanisms. Comprehensive analyses ultimately identified NUP98 and TOP1 as key genes, with stable chondrocytes (HomCs) as the key cell type.

NUP98, a nuclear pore protein encoded by the NUP98 gene, is a key component of the nuclear pore complex (NPC) and primarily mediates the transport of materials between the nucleus and cytoplasm, such as RNA and proteins. Through interactions with other nuclear pore proteins, NUP98 plays a critical role in processes like mRNA transport and nuclear protein export (53, 54). Additionally, NUP98 is involved in cell cycle regulation and gene expression. Dysregulation of its function can influence cell fate (proliferation, differentiation, apoptosis), and mutations, rearrangements, or fusions of this gene are closely related to the development and progression of acute leukemias and other cancers (55–58). Recent studies have integrated multi-omics data and machine learning algorithms to identify energy metabolism-related OA subtypes and have demonstrated that NUP98 and RPIA can serve as diagnostic biomarkers and potential therapeutic targets for OA (59). The convergence between these findings and our current results-demonstrating NUP98’s significant downregulation in OA tissues and its association with SUMOylation pathways-provides compelling evidence for its dual role: as a nuclear transport regulator maintaining chondrocyte homeostasis, and as a SUMOylation substrate potentially involved in OA pathogenesis. This mechanistic insight not only advances our understanding of post-translational modifications in joint degeneration but also opens new avenues for developing NUP98-targeted diagnostic strategies and precision therapies for OA.

TOP1 encodes DNA topoisomerase I (TOP1), which regulates DNA topology and maintains genomic stability by catalyzing transient DNA single-strand breaks and religation. Its core function is to resolve DNA superhelical tension, ensuring faithful DNA replication, transcription, and repair (60). Although TOP1 dysregulation is well-characterized in multiple cancers (61, 62), recent studies reveal its multifaceted role in OA pathogenesis: The circ0072568/miR-382-5p/TOP1 regulatory axis has been demonstrated to mediate chondrocyte inflammatory responses and extracellular matrix degradation (63), while evidence identifies TOP1 as a potential OA diagnostic biomarker (64). Our study extends this paradigm by uncovering, for the first time, TOP1’s association with protein SUMOylation in OA, suggesting its dual mechanistic roles in genome maintenance and post-translational regulation of joint homeostasis. These findings not only consolidate TOP1’s diagnostic utility but also provide novel therapeutic entry points for targeting TOP1-associated SUMOylation networks in OA.

The significant co-enrichment of both NUP98 and TOP1 in the mRNA processing pathway across bone, synovium, and cartilage provides the missing functional link. This suggests that their synergistic role in OA may stem from co-regulating the maturation and metabolism of mRNAs, a process fundamental to cellular homeostasis. This finding logically integrates their established individual roles: NUP98, as a nucleoporin, facilitates the nuclear export of processed mRNA (65), while TOP1, by resolving DNA supercoiling during transcription, is pivotal for mRNA synthesis (66). Their concomitant dysregulation in OA could therefore disrupt this critical axis of gene expression governance, contributing to joint-wide pathology. The stronger enrichment signal for TOP1 may indicate its more upstream position in this coordinated network.

We identified 14 distinct immune cell subsets with significant abundance differences between OA and control groups, including activated/resting dendritic cells (aDCs/rDCs), eosinophils, M1/M2 macrophages, monocytes, neutrophils, activated/resting NK cells, CD8^+^ T cells, naive CD4^+^ T cells, and γδ T cells. These findings reveal a complex immune dysregulation within the OA joint microenvironment. M1 macrophages (pro-inflammatory) are significantly enriched in OA synovium (67) and directly trigger chondrocyte apoptosis and matrix metalloproteinase (MMP) expression via TNF-*α*, IL-1β, and other cytokines. While M2 macrophages (reparative) exhibit anti-inflammatory potential, their aberrant expansion may drive fibrotic repair, synovial hyperplasia, and osteophyte formation (68). Activated DCs (aDCs), as potent antigen-presenting cells, amplify local inflammation by activating T-cell responses in OA synovial fluid (69). NUP98 shows significant positive correlations with pro-inflammatory aDCs and M1 macrophages, suggesting its nuclear transport function may regulate immune cell activation. TOP1 positively correlates with reparative M2 macrophages and monocytes, implicating its DNA helicase activity in immune cell differentiation trajectories. Collectively, NUP98 and TOP1 may shape the OA-specific pro-inflammatory microenvironment through modulation of macrophage polarization and DC maturation—thereby accelerating joint degeneration.

We constructed a competing endogenous RNA (ceRNA) network, identifying 14 miRNAs and 18 lncRNAs targeting key genes (NUP98/TOP1). Functionally validated interactions include: hsa-miR-382-5p exacerbates OA chondrocyte apoptosis by suppressing TOP1 expression (63). hsa-miR-26b-5p exerts chondroprotective effects via dual mechanisms: direct anti-inflammatory activity and preservation of TOP1-mediated genomic stability (70). hsa-miR-18a-3p (upregulated in OA synovial fluid) (71) directly targets NUP98, potentially amplifying inflammatory cascades by impairing nucleocytoplasmic transport of transcription factors (e.g., NF-κB). These findings delineate two core regulatory hubs in OA pathogenesis: The TOP1 network (miR-382-5p/TOP1-DNA repair; miR-26b-5p/TOP1-anti-inflammation) maintains genomic integrity. The NUP98 network (miR-18a-3p/NUP98-nuclear signaling) governs inflammatory balance. Collectively, they orchestrate chondrocyte homeostasis, providing a molecular basis for RNA-targeted therapeutics.

High-throughput screening predicted 44 NUP98-targeting agents and 38 TOP1-targeting agents, with 8 compounds exhibiting dual-targeting potential: digitoxigenin, anisomycin, luteolin, irinotecan, ZINC, (−)-epigallocatechin gallate (EGCG), daunorubicin, and ouabain. These polypharmacological agents demonstrate compelling therapeutic potential through distinct yet complementary mechanisms: Anisomycin—a known JNK/p38 activator—suppresses MMP-13 expression in OA chondrocytes by inducing protective autophagy (72, 73); luteolin potently attenuates IL-1β-driven inflammation by inhibiting nuclear translocation of NF-κB (74), an effect potentially potentiated through interaction with NUP98-mediated nucleocytoplasmic trafficking. Notably, EGCG restores mitochondrial function via SIRT1-PGC-1*α* activation (75) while concurrently stabilizing DNA through TOP1 interaction, thereby conferring dual efficacy. These findings highlight the therapeutic advantage of multi-target agents such as anisomycin and EGCG, which simultaneously address the triad of core OA pathologies: genomic stability (via TOP1 modulation), nuclear transport efficiency (through NUP98 targeting), and inflammatory pathway regulation, offering an integrated strategic approach to OA therapy.

Single-cell transcriptomics deconstructs articular tissue heterogeneity to precisely identify homeostatic chondrocytes (HomCs) as pivotal effector cells driving osteoarthritis (OA) progression. Cell communication network analysis reveals that HomCs propel joint degeneration through multiple signaling axes, including macrophage recruitment and the establishment of a TNF-α/IL-1β-NF-κB inflammatory positive feedback loop, which activates synovial fibroblasts to release MMP3/9 among other effectors (76). Crucially, pseudotime trajectory analysis demonstrates that OA triggers the collapse of the HomCs differentiation program. Collectively, these findings establish a cascade model of HomCs homeostatic disruption in OA: the lipotoxic microenvironment initiates metabolic-epigenetic reprogramming, followed by NUP98-mediated nucleocytoplasmic transport disorders and TOP1-driven genomic instability, and finally pathological states solidified through cell communication networks (77, 78). This model unveils novel interventional strategies targeting the FAO-AMPK axis or APOE-LRP1 signaling to restore chondral homeostasis.

This study integrates multi-omics data with computational biology approaches to systematically identify, for the first time, two SUMOylation-associated hub genes (NUP98, TOP1) and a core effector cell subpopulation (homeostatic chondrocytes, HomCs) in OA pathogenesis. We elucidate their synergistic molecular mechanism in driving OA progression via dysregulation of nucleocytoplasmic transport (NUP98), genomic stability (TOP1), and intercellular communication networks (HomCs-mediated tripartite signaling axes). The diagnostic model, ceRNA regulatory network, and drug repositioning strategy derived from these findings provide a novel theoretical framework and translational pathway for OA early warning and targeted therapy.

However, this study has limitations: Bioinformatics constraints: Conclusions are contingent on public dataset quality and algorithmic assumptions, with potential confounding by batch effects or false-positive results. Functional validation needs: Mechanistic roles of hub genes and HomCs require further validation via gene editing (e.g., CRISPR-Cas9) and organoid models. Therapeutic candidate evaluation: *In vivo* efficacy and target specificity of predicted compounds (e.g., anisomycin, EGCG) necessitate assessment in animal models. Future work will focus on establishing OA-humanized tissue chips to validate the NUP98/TOP1-HomCs regulatory axis and investigating the fine-tuning role of SUMOylation in this pathway to advance clinical translation of precision interventions.

## Data Availability

The original contributions presented in the study are included in the article/supplementary material, further inquiries can be directed to the corresponding author.

## Footnotes

1
https://www.ncbi.nlm.nih.gov/geo/

2
https://string-db.org

3
https://rdrr.io/github/yikeshu0611/ggDCA/

4
https://www.gsea-msigdb.org/gsea/index.jsp

5
https://genemania.org/

6
https://mirtarbase.cuhk.edu.cn

7
http://www.mirdb.org

8
https://www.mirnet.ca/upload/MirUploadView.xhtml

9
https://maayanlab.cloud/Enrichr/

## Supplementary material

SUPPLEMENTARY TABLE S1 List of SUMOylation-related genes.

SUPPLEMENTARY TABLE S2 Primary antibodies used in immunohistochemical analysis.

SUPPLEMENTARY TABLE S3 Primer sequences for RT-qPCR.

SUPPLEMENTARY TABLE S4 Primary antibodies used in Western blot analysis.

SUPPLEMENTARY TABLE S5 Detailed results of GO enrichment analysis.

SUPPLEMENTARY TABLE S6 Results of KEGG pathway enrichment analysis.

SUPPLEMENTARY TABLE S7 Gene set enrichment analysis results for NUP98.

SUPPLEMENTARY TABLE S8 Gene set enrichment analysis results for TOP1.

SUPPLEMENTARY TABLE S9 Results of immune cell correlation analysis.

SUPPLEMENTARY TABLE S10 Correlation analysis between key genes and immune cells.

SUPPLEMENTARY TABLE S11 List of shared microRNAs.

SUPPLEMENTARY TABLE S12 List of long non-coding RNAs.
