# Supplementary figures and images for: Identification and validation of SUMOylation-related key genes for osteoarthritis through integration of single-cell, bulk RNA sequencing and animal model experiments
Source: Front Med (Lausanne). 2026 Mar 31;13:1779874. doi: 10.3389/fmed.2026.1779874 (PMC13076273; doi:10.3389/fmed.2026.1779874)

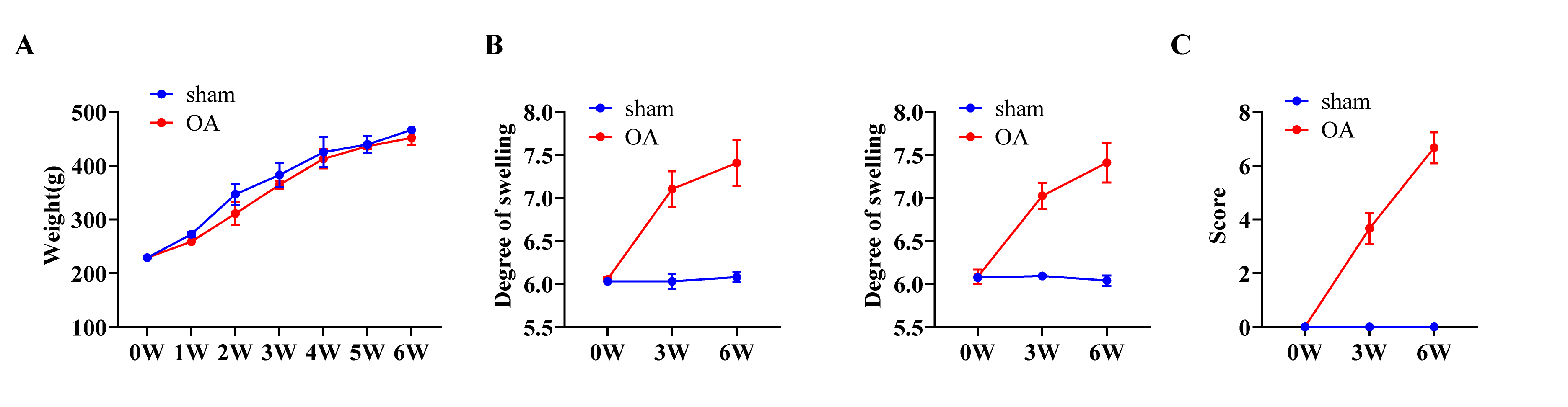

Supplement: Supplementary file 2 [file Supplementary_file_1.zip › Figure S5.tif]

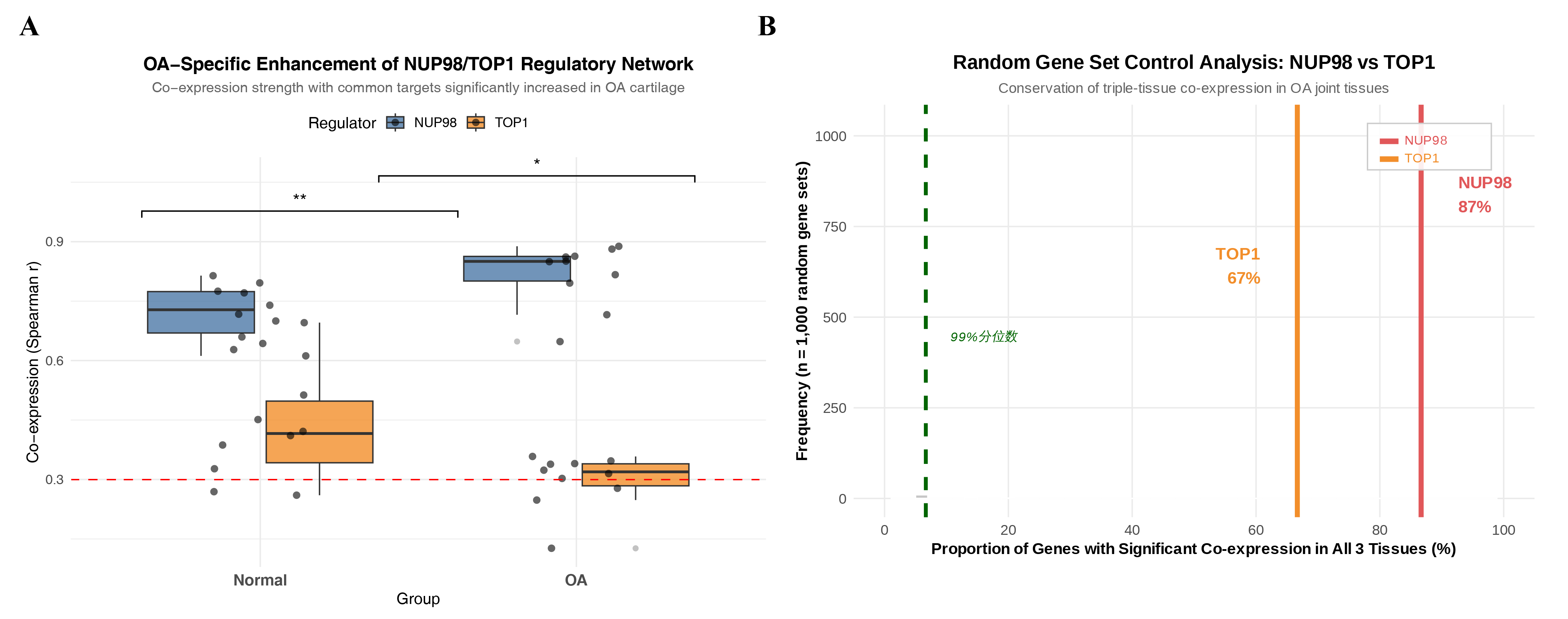

Supplement: Supplementary file 2 [file Supplementary_file_1.zip › Figure S1.tif]

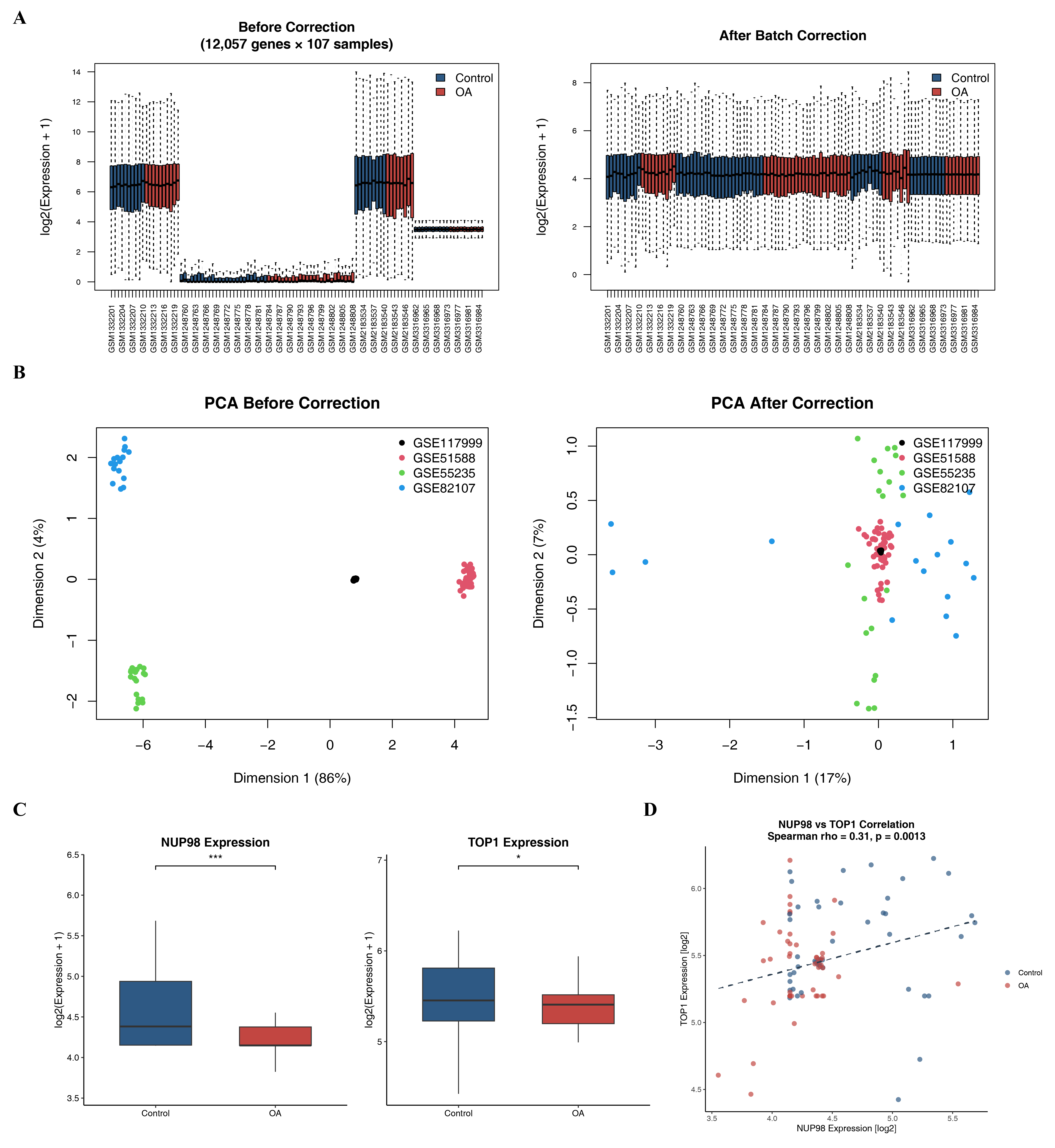

Supplement: Supplementary file 2 [file Supplementary_file_1.zip › Figure S2.tif]

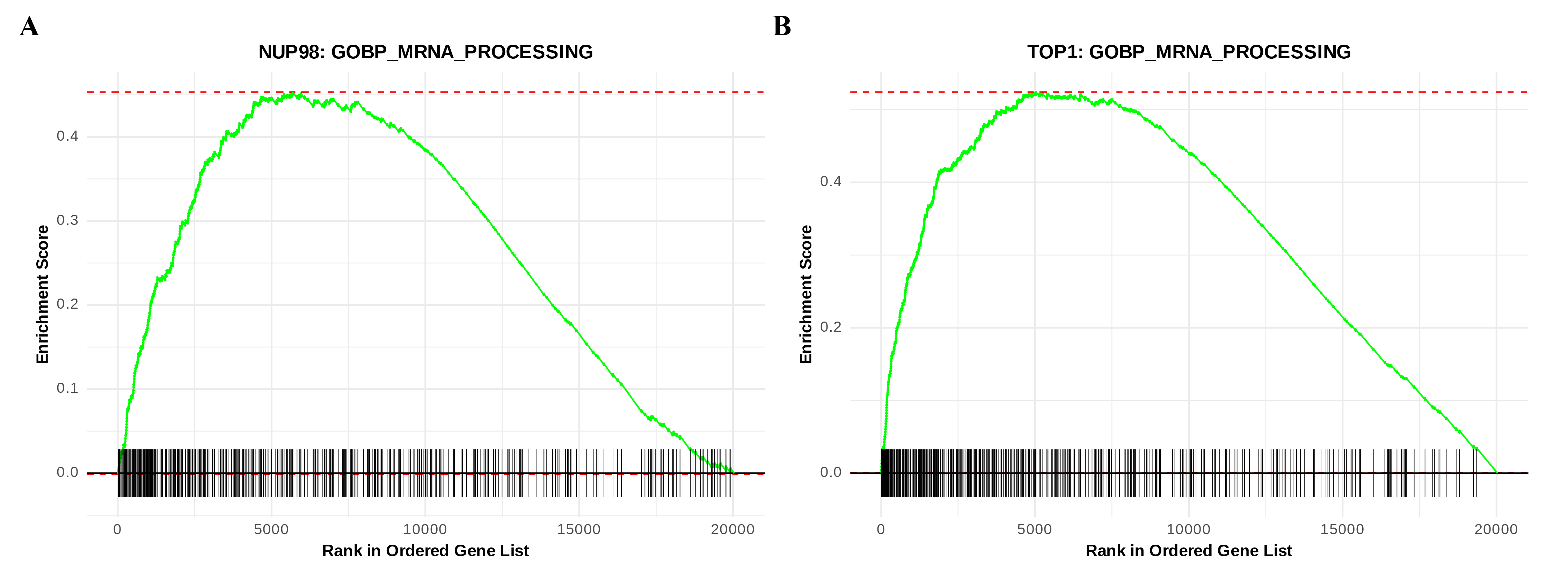

Supplement: Supplementary file 2 [file Supplementary_file_1.zip › Figure S3.tif]

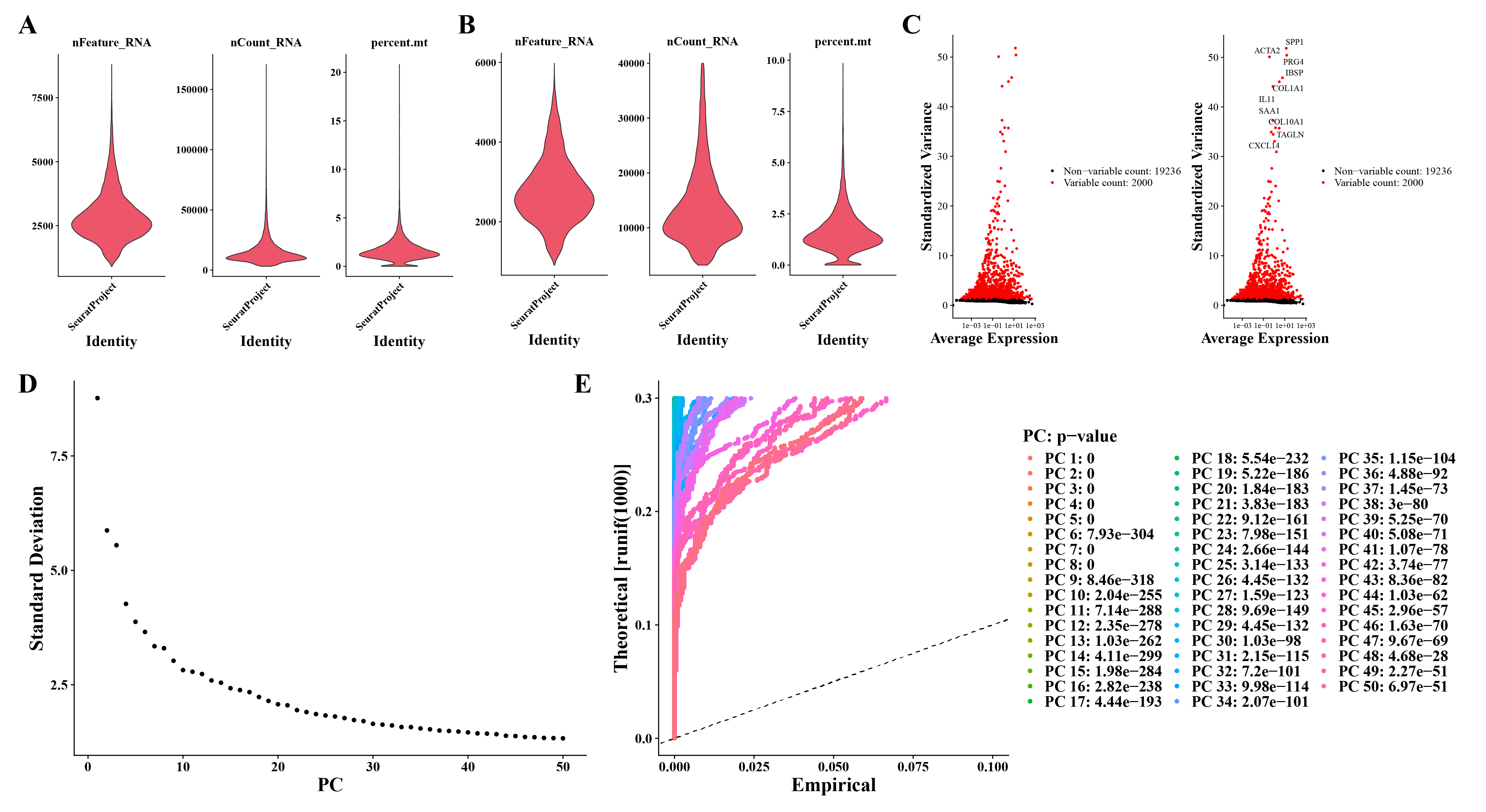

Supplement: Supplementary file 2 [file Supplementary_file_1.zip › Figure S4.tif]
